# Chemogenetic Activation of G_12_ Signaling Thickens the Epidermis With Enhanced Barrier Function

**DOI:** 10.1096/fba.2026-00042

**Published:** 2026-07-31

**Authors:** Nozomi Kamakura, Natsumi Hirai, Kaito Arai, Yaxin Du, Toshiaki Kogame, Yusuke Ohno, Akio Kihara, Kenji Kabashima, Asuka Inoue

**Affiliations:** ^1^ Graduate School of Pharmaceutical Sciences Kyoto University Kyoto Japan; ^2^ Graduate School of Pharmaceutical Sciences Tohoku University Sendai Japan; ^3^ Department of Dermatology, Graduate School of Medicine Kyoto University Kyoto Japan; ^4^ Faculty of Pharmaceutical Sciences Hokkaido University Sapporo Japan; ^5^ Faculty of Pharmacy Juntendo University Chiba Japan

**Keywords:** DREADD, epidermal thickening, G_12_ signaling, GPCR, skin barrier

## Abstract

The epidermis provides the body's outermost barrier, yet how G‐protein‐coupled receptor (GPCR) signaling via the G_12/13_ family regulates epidermal homeostasis in vivo remains unclear. Here, we selectively activated G_12_ signaling in keratinocytes using a chemogenetic strategy. Activation of a G_12_‐coupled designer receptor (G_12_D) in mouse epithelial cells induced pronounced epidermal thickening while preserving stratified architecture and avoiding overt inflammatory skin changes. This thickening was accompanied by increased Ki67‐positive cells, expansion of keratin 10‐ and filaggrin‐positive layers, and transcriptomic upregulation of genes related to keratinocyte differentiation, keratinization, and epidermal barrier function. Functionally, G_12_D activation strengthened barrier performance, as shown by blunted transepidermal water loss responses to mechanical barrier disruption. Although alarmin‐related genes were upregulated, cytokine analyses indicated only modest inflammatory changes. Pharmacologic inhibition of TYK2 (deucravacitinib) partially reduced G_12_D‐driven epidermal thickening, whereas mTORC1 inhibition (rapamycin) produced a stronger suppressive effect, suggesting that the mTORC1‐dependent keratinocyte response is a major driver of this phenotype, with a moderate TYK2‐dependent component. Together, these findings identify epidermal G_12_ signaling as a regulator that promotes “non‐pathological” epidermal thickening coupled to enhanced barrier function, supporting G_12_‐coupled GPCRs as potential therapeutic entry points for barrier‐compromised skin disorders.

## Graphical abstract

In this study, we investigated the role of G_12_ signaling in the epidermis using a keratinocyte‐specific chemogenetic approach. Activation of G_12_ signaling promoted keratinocyte differentiation and enhanced epidermal barrier function by upregulating filaggrin, claudin‐1, and ceramide. This activation also induced epidermal thickening through mTORC1 and TYK2, with mTORC1 contributing more strongly than TYK2.

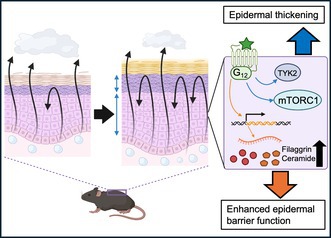

## 1 Introduction

The skin, and particularly the epidermis, constitutes the body's outermost barrier, protecting against environmental insults and limiting transepidermal water loss. Maintaining this barrier requires a tightly balanced program of keratinocyte proliferation and differentiation [1]. Perturbations in epidermal turnover can shift this balance and lead to epidermal thickening [2, 3], which is frequently associated with inflammatory skin disorders and barrier impairment [4, 5]. However, epidermal thickening is not invariably pathological: when stratification and differentiation remain intact, an expanded epidermis can increase the number of barrier layers and potentially strengthen barrier function. Signaling pathways that drive such “nonpathological” thickening remain incompletely defined. Receptor tyrosine kinases (RTKs) such as EGFR provide one example—retinoids can upregulate epidermal growth factor (EGF) and promote epidermal thickening via EGFR signaling [6]. Beyond RTKs, additional membrane receptor families have begun to emerge as candidate regulators of epidermal growth and barrier homeostasis.

Among these, G‐protein‐coupled receptors (GPCRs) are particularly attractive because they are broadly expressed in skin and are highly druggable. GPCRs couple to four major classes of heterotrimeric G proteins (G_s_, G_i_, G_q_, and G_12_) to elicit distinct intracellular responses. Various studies have reported associations between GPCR signaling and epidermal thickening, as well as keratinocyte proliferation and differentiation. For example, activation of the prostaglandin E2 receptor EP2, a G_s_‐coupled GPCR, enhances keratinocyte proliferation, whereas activation of the cannabinoid receptor CB1, a G_i_‐coupled GPCR, and histamine receptor H1R, a G_q_‐coupled GPCR, suppresse keratinocyte differentiation [7, 8, 9]. More recently, activation of the G_12_‐coupled GPCRs lysophosphatidic acid receptor 1 (LPAR1) and LPAR5 has been shown, in a three‐dimensional culture system, to promote keratinocyte differentiation [10]. Despite these advances, the in vivo consequences of selectively engaging epidermal G_12_ signaling remain largely unknown.

Chemogenetic approaches using Designer Receptors Exclusively Activated by Designer Drugs (DREADDs)—engineered GPCRs—enable spatiotemporally controlled activation of defined G‐protein pathways in specific cell types. The most widely used DREADDs are muscarinic acetylcholine receptor variants that are unresponsive to acetylcholine but can be selectively activated by the synthetic ligand clozapine N‐oxide (CNO) [11]. In our previous studies, we generated a G_12_‐coupled DREADD (G_12_D) from a G_q_‐coupled DREADD and established transgenic mice in which G_12_D expression is induced in a Cre‐dependent manner [12, 13]. Of the two members of the G_12_ class G proteins, G_12_ is strongly activated by G_12_D, whereas G_13_ is only weakly coupled [14]. This platform has elucidated roles for G_12_ signaling in multiple tissues, including adipose tissue, liver, and POMC neurons, and provides a direct strategy to interrogate G_12_ signaling in additional contexts such as the epidermis [14, 15].

Here, we used keratinocyte‐specific G_12_D expression to define the role of epidermal G_12_ signaling in vivo. Using K14‐G_12_D mice, we show that activation of epidermal G_12_ signaling induces epidermal thickening and enhances skin barrier function. Mechanistically, the thickening response is mediated by mTORC1 and TYK2. Together, these findings identify epidermal G_12_ signaling as a pathway that can promote barrier‐supportive epidermal expansion and suggest G_12_‐coupled GPCRs as potential therapeutic entry points for barrier‐compromised skin diseases.

## 2 Methods and Materials

### 2.1 Animal Experiments

Mice were maintained according to the Guidelines for Animal Experimentation of Tohoku University and in compliance with the Ethical Regulations of Kyoto University, and the protocol was approved by the Institutional Animal Care and Use Committee at Tohoku University (permission number: 2025Ph‐006) and the Animal Care and Experimentation Committee of Kyoto University (permission number: 25‐81). All mice were kept on a 12‐h light/12‐h dark cycle (09:00–21:00 or 08:00–20:00) at a controlled temperature (23°C) and were given free access to water and food. All experiments were conducted using female mice at 8–11 weeks of age.

Wax depilation was performed as follows. Female mice aged 8–9 weeks were anesthetized with mixed three‐agent anesthesia (10 mL/kg). The dorsal skin was depilated by applying depilation wax (SURGI WAX) twice. After the procedure, the mice were placed on a 42°C hot plate to recover from anesthesia.

K14‐G_12_D and control mice were intraperitoneally administered CNO (10 mg/kg/day) for 10 days. Saline was used as the vehicle for CNO. In inhibitor treatment experiments, mice received CNO together with each inhibitor as follows. For Sotyktu treatment, mice received intraperitoneal administration of CNO (10 mg/kg/day) and oral administration of Sotyktu (10 mg/kg/day) for 10 days. A 0.5% methylcellulose solution was used as the vehicle for Sotyktu. For rapamycin treatment, a mixed solution containing CNO (final dose, 10 mg/kg/day) and rapamycin (final dose, 3 mg/kg/day) was administered intraperitoneally. DMSO was used as the vehicle for rapamycin.

### 2.2 Materials

CNO was synthesized at the International Institute for Integrative Sleep Medicine (WPI‐IIIS), University of Tsukuba as described previously [13]. Sotyktu was purchased from Bristol Myers Squibb. Rapamycin (cat. HY‐10219) was purchased from MedChem Express.

### 2.3 Generation of Epidermis‐Specific G_12_D‐Expressing Mice

*ROSA26*‐*LSL*‐*G12D*‐*IRES*‐*GFP* mice were generated as described previously [13]. Epidermis‐specific G_12_D‐expressing mice (K14‐G_12_D mice) were generated by mating *ROSA26*‐*LSL*‐*G12D*‐*IRES*‐*GFP* mice with Krt14‐Cre mice (The Jackson Laboratory; stock no. 018964). All experiments were carried out with female littermates because male mice tend to fight each other, making it difficult to observe the depilated dorsal skin. *ROSA26*‐*LSL*‐*G12D*‐*IRES*‐*GFP* littermate mice that lacked the Krt14‐Cre transgene were used as a control in all experiments.

### 2.4 RNA Isolation and Quantitative RT‐PCR

Total RNA from skin tissue was isolated using Isogen II (Nippon Gene, Japan) and then reverse‐transcribed with a High‐Capacity cDNA Reverse Transcription Kit (Applied Biosystems) according to the manufacturer's instructions. The cDNA product was amplified by qRT‐PCR performed with TB Green Premix Ex Taq II (Tli RNaseH Plus) (Takara Bio) and monitored by ABI Prism 7300 (Applied Biosystems). Gene expression was normalized to the expression of GAPDH or HPRT. The PCR primer sequences are listed in Table S1.

### 2.5 RNA‐Seq and Analysis

For RNA‐seq, total RNA was extracted from whole dorsal skin samples collected from K14‐G_12_D and control mice (*n* = 3) using Isogen II (Nippon Gene) and the RNeasy Plant Mini Kit (Qiagen) following the manufacturer's protocol. RNA‐seq libraries were then prepared from these samples. Poly(A)^+^ RNA was isolated using the NEBNext Poly(A) mRNA Magnetic Isolation Module (E7490, New England Biolabs), and strand‐specific libraries were constructed using the NEBNext Ultra II Directional RNA Library Prep Kit (E7760, New England Biolabs). Sequencing was performed on an Illumina NovaSeq X Plus in paired‐end 150‐bp mode. Base calling and demultiplexing were performed using BCL Convert v4.4.6. Trimmed reads were quantified against the mouse reference transcriptome ( *Mus musculus* GRCm39, Ensembl release 115) using Salmon quant in Galaxy. Transcript‐level abundance estimates were summarized to gene‐level counts using the corresponding GTF annotation file, and differential gene expression analysis was performed using DESeq2 in Galaxy. Gene expression was calculated as transcripts per kilobase million mapped sequence reads. Gene ontology (GO) enrichment analysis was performed using g:Profiler (version e114_eg62_p19_27110d83; database updated on March 20, 2026). Upregulated differentially expressed genes were identified using DEseq2, and genes with an adjusted *p* < 0.05 and |log_2_| > 1 were used for enrichment analysis. GO biological process terms were analyzed, and the top enriched terms were displayed. Gene set enrichment analysis (GSEA) was performed using GSEA software from the Broad Institute to identify pathways enriched in bulk RNA‐seq data from control and K14‐G_12_D mice. A normalized gene expression matrix was used as input, and gene sets with a false discovery rate (FDR) < 0.25 and nominal *p* < 0.05 were considered significantly enriched.

### 2.6 Histology, Immunohistochemistry, and Immunofluorescence

Skin tissues were fixed in 4% paraformaldehyde for 24 h. Preparation of sections and immunohistochemistry and Masson's trichrome staining were performed by the Pathology section of Biomedical Research Core of Tohoku University Graduate School of Medicine. In the immunohistochemistry analysis, an anti‐HA antibody (cat. 3724, Cell Signaling Technology) was used as the primary antibody, and Histofine Simple Stain Max‐PO (cat. 414341, Nichirei Biosciences) served as the secondary antibody. 3,3′‐diaminobenzidine (DAB, Dojindo Laboratories) was used for colorimetric detection. In Masson's trichrome staining, hematoxylin was used to stain nuclei; 0.75% Orange G to stain erythrocytes; Ponceau/acid fuchsin to stain muscle fibers and cytoplasm; and aniline blue to stain collagen fibers. Mouse skin samples were excised, immediately transferred to 4% paraformaldehyde in PBS, fixed overnight, and rinsed. They were then dehydrated through a series of graded ethanols and xylene, and embedded in paraffin. Sections (5 μm) were processed with a microtome. Hematoxylin and eosin (H&E) staining was performed according to a standard protocol. Bright‐field images of the stained tissue sections were taken with Zeiss Axio Imager equipped with visualix V310B and Olympus LV200 LUMINOVIEW with an objective (UPlanSApo, ×20 and ×60/1.40, Olympus), a 36‐mm imaging lens (Magnification ×0.2), and a color CMOS camera (Alvium 1800 U‐234c, Allied Vision) for high‐magnification images. Toup View (ToupTek Photonics) was used for quantification. For immunofluorescence staining, paraffin‐embedded skin sections were incubated overnight at 4°C with primary antibodies against keratin 10 (1:500, cat. 905403, BioLegend), keratin 14 (1:200, cat. 905303, BioLegend), filaggrin (1:1000, cat. PRB‐417P, Covance), Ki67 (1:200, cat. ab16667, Abcam), and CD3 (1:100, cat. Ab16669, Abcam). The signal was also visualized using the Opal 3‐Plex Manual Kit (Akoya Biosciences), which allows simultaneous detection of multiple targets in the same image. Secondary antibodies (undiluted), ImmPRESS Reagent (MP‐7401, MP‐7404, Vector Laboratories), EnVision+ System‐HRP Labeled Polymer (K4001, Dako), and Fluorophores Opal 570, Opal 620, Opal 670 and Opal690 were used, and the sections were counterstained with Spectral DAPI. All immunofluorescence images were collected from each stained slide on a BX43 microscope (Olympus) equipped with a Mantra Quantitative Pathology Imaging System (PerkinElmer). Image acquisition was performed using Mantra/Mantra Snap software (version 1.0.3.), and spectral unmixing was performed using inForm software (version 2.6.0; PerkinElmer). For quantification of Ki67‐positive cells, equivalent regions of interest (ROI) were selected, and Ki67‐positive nuclei within the epidermis were counted manually. For quantification of CD3‐positive cells, a rectangular ROI of identical size was manually placed in each image so that it extended from the outer surface of the epidermis into the superficial dermis. CD3‐positive cells within the ROI were detected using the Positive Cell Detection algorithm of QuPath (version 0.7.0.) with identical detection parameters applied to all images.

### 2.7 Lipid Extraction and Analysis by LC/MS/MS

Skin tissue (10 mg) was transferred to a tube containing zirconia beads and suspended in 450 μL of chloroform/methanol/12 M formic acid (100:200:1, v/v). As internal standards, the following deuterium‐labeled ceramides were added to the sample: 1 nmol *N*‐palmitoyl(*d*_9_) d‐*erythro*‐sphingosine (*d*_9_‐C16:0 NS), 2 nmol *N*‐palmitoyl(*d*_9_) dihydrosphingosine (*d*_9_‐C16:0 NDS), 0.2 nmol *N*‐palmitoyl(*d*_9_) d‐*ribo*‐phytosphingosine (*d*_9_‐C16:0 NP), and 0.5 nmol *N*‐(2′‐(*R*)‐hydroxypalmitoyl(*d*_9_)) d‐*erythro*‐sphingosine (*d*_9_‐C16:0 AS) (all from Avanti Research, Alabaster, AL, USA). The skin samples were disrupted using a Micro Smash MS‐100 (TOMY Seiko, Tokyo, Japan) by vigorous shaking (4500 rpm, 1 min, 4°C) and then centrifuged (20,000*g*, 3 min, room temperature). The resulting supernatant was recovered, mixed with 150 μL of chloroform and 270 μL of water, and centrifuged to separate the phases. The lower organic phase was collected, dried, and dissolved in 1000 μL of chloroform/methanol (1:2, v/v). The lipid samples were diluted 200‐fold, and 5 μL was subjected to LC–MS/MS analysis. Ceramides were detected and quantified using UPLC coupled with an ESI tandem quadrupole mass spectrometer (Xevo TQ‐S; Waters, Milford, MA). LC separation and ionization by ESI condition were performed as previously described [16, 17]. Each ceramide species was detected in the MRM mode using *m/z* values of precursor ions (Q1) and product ions (Q3) and collision energies listed in Table S2. Major molecular species of ceramides, including NS, NDS, NP, EOS, OS, and BS, were quantified, covering approximately 97% of the total ceramides in the mouse stratum corneum [17]. Ceramides were quantified by calculating the ratio of the peak area of each ceramide species compared with that of the deuterium‐labeled ceramide corresponding to each ceramide class. EOS, OS, and BS ceramides were quantified using the deuterium‐labeled AS that have common long‐chain base structures. MassLynx software (Waters) was used for data analysis.

### 2.8 Measurement of Protein Amount

Stratum corneum was collected by tape stripping. 2 or 3 or 4 consecutive tape strips were collected from the same area. The tapes were cut into small pieces, and proteins were extracted by heating them at 60°C for 2 h in a buffer containing 0.1 M NaOH and 1% SDS. The protein concentration was then quantified using the Pierce BCA Protein Assay Kit (Thermo Fisher Scientific).

### 2.9 Measurement of Transepidermal Water Loss

Mice were denuded by wax depilation and tape stripping prior to measurements. Transepidermal water loss (TEWL) was measured with an evaporimeter (ServoMed, Stockholm, Sweden). Measurements were performed at 24°C and 46% relative humidity.

### 2.10 Flow Cytometry Analysis

Flow cytometry experiments were performed on whole dorsal skin cells collected from CNO‐treated control and K14‐G_12_D mice. For collecting whole dorsal skin cells, whole dorsal skin was treated with liberase (cat. 05401020001; Roche). The collected cells were blocked with mouse Fc Block (1:200, anti CD16/CD32 antibody; cat. 553142, BD Biosciences) and incubated with eFlour 506‐conjugated FVD (1:2000; cat. 65‐0866‐14; Invitrogen) and CD11c (1:300, cat. 14‐0114‐82; eBioscience) for 10 min on ice. Then the cells were incubated with the following antibodies for 20 min on ice: BUV395‐conjugated CD45 (1:1000, cat. 564279; BD Biosciences), BV605‐conjugated MHC II (1:1000, cat. 562845; BD Biosciences), BUV737‐conjugated SA (1:400, cat. 612775; BD Biosciences), PE‐Cy7‐conjugated TCRβ (1:200, cat. 109221; BioLegend), APC‐Cy7‐conjugated CD11b (1:200, cat. 561039; BD Biosciences), APC‐conjugated γδTCR (1:200, cat. 118115; BioLegend), BV786‐conjugated CD64 (1:100, cat. 744739; BD Biosciences). For CD64 staining, an isotype‐matched control antibody (1:100, BV786‐conjugated Rat IgG2a, cat. 563335; BD Biosciences) was used as a negative control to assess background and nonspecific staining in whole‐skin FACS analysis. Samples were acquired on a BD LSRFortessa Flow Cytometer (BD Biosciences). Flow cytometry data were analyzed using FlowJo software (version 10.10.1).

### 2.11 Quantification of skin wrinkles

For each gross image of the dorsal skin, five horizontal lines were drawn. The number of intersections between each line and the wrinkles was counted and used as the wrinkle count.

### 2.12 Statistics

Data were analyzed by using the Prism 10 software (GraphPad) and Microsoft Excel (Microsoft) software. All data were expressed as means ± SEM for the indicated number of independent experiments or observations. Statistical significance was tested by the unpaired Student's *t*‐test (two‐tailed) or by the two‐way ANOVA followed by the Sidak's post hoc tests, as appropriate. *p* < 0.05 were considered statistically significant.

## 3 Results

### 3.1 Epidermal G_12_D Activation Induces Epidermal Thickening

**FIGURE 1:**
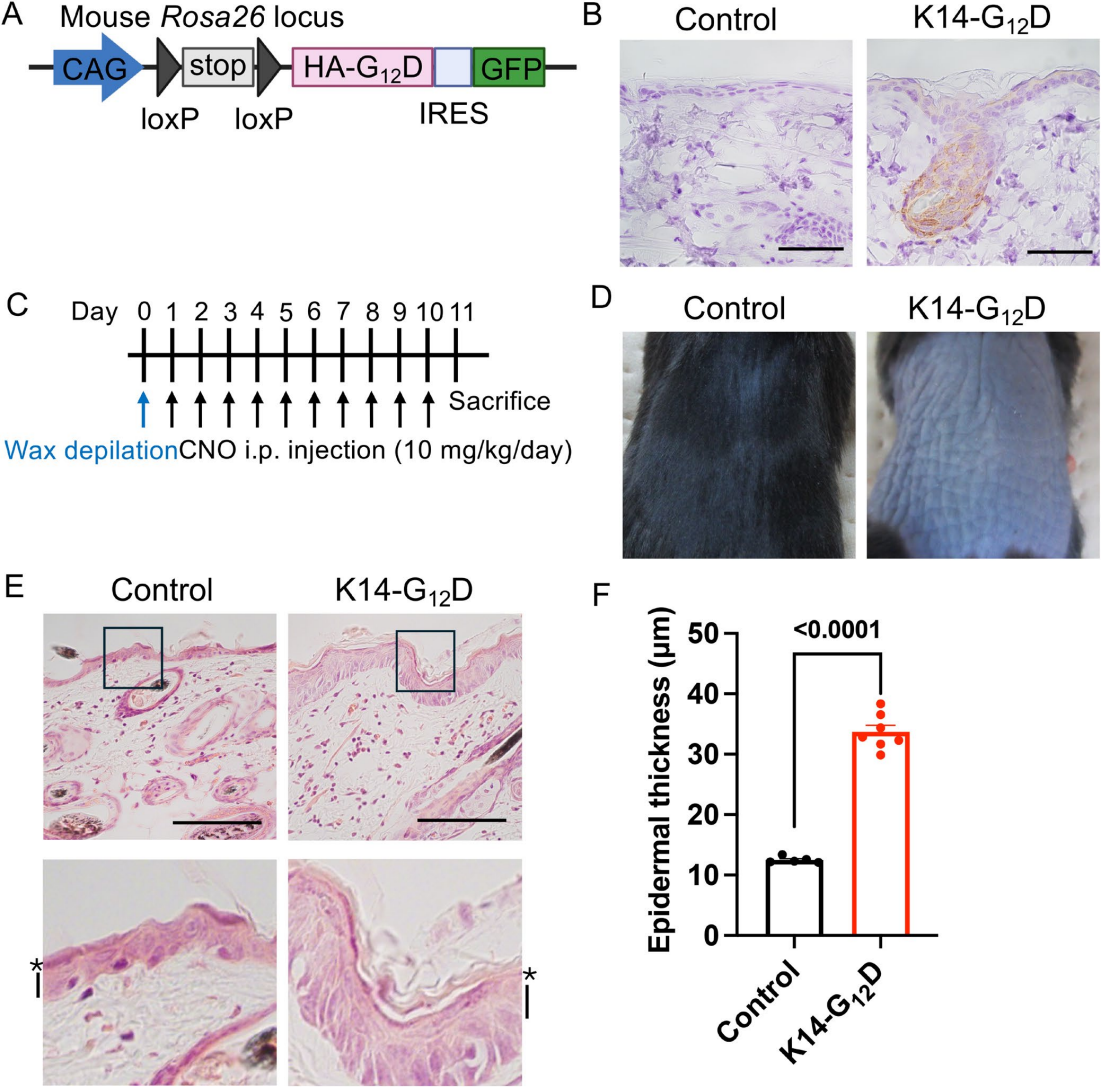
Epidermal G_12_D activation induces epidermal thickening. (A) Schematic diagram of the *ROSA26*‐*LSL*‐*G12D*‐*IRES*‐*GFP* allele. CAG, CAG promoter; GFP, green fluorescent protein; HA‐G_12_D, HA epitope tag‐fused G_12_D, IRES: internal ribosome entry site; Stop, a stop codon cassette; (B) Immunostaining showing epidermis‐specific G_12_D expression in K14‐G_12_D and control mice. (C–E) Effect of G_12_D activation on epidermal morphology. Female K14‐G_12_D mice and control mice were depilated, followed by daily i.p. injections of CNO (10 mg/kg/day) for 10 days. CNO injections were started at 8 weeks of age. (C) Protocol for CNO administration and wax depilation. (D and E) Representative appearance of dorsal skin (D) and hematoxylin and eosin (HE) staining of dorsal skin sections (E). Asterisks indicate the stratum granulosum, and black lines indicate the stratum spinosum. Scale bars: 100 μm. (F) Quantification of epidermal thickness shown in (E) (*n* = 5–7 per group). Values represent mean ± SEM. Data were analyzed by the two‐tailed Student's *t*‐test.

We generated K14‐G_12_D mice that express G_12_D specifically in the epidermis. We crossbred the *ROSA* *26*‐*LSL*‐*G12D*‐*IRES*‐*GFP* (LSL‐G_12_D) mice with Krt14‐Cre mice, which express Cre recombinase in the keratin 14‐positive epithelial cells including keratinocytes [18]. Crossing homozygous LSL‐G_12_D mice and heterozygous Krt14‐Cre mice resulted in a 1:1 Mendelian ratio of mice carrying both the K14‐Cre cassette and the LSL‐G_12_D cassette (K14‐G_12_D mice) and littermates carrying only the LSL‐G_12_D cassette, which were used as a control group. Since the *Krt14* promoter drives Cre expression in the basal layer keratinocytes, G_12_D is expected to be expressed throughout the entire epidermis due to irreversible Cre‐mediated recombination [19, 20]. We analyzed the protein expression of G_12_D in skin specimens by immunohistochemistry using an anti‐HA tag antibody targeting the N‐terminus of G_12_D (Figure 1A). As expected, HA immunoreactivity was detected in epithelial regions, including the interfollicular epidermis and follicular epithelium, of K14‐G_12_D mice, whereas no immunostaining signal was detected in the control mice (Figure 1B and Figure S1).

To examine the effect of G_12_D activation in the epidermis, we administered a designer ligand, CNO, to the K14‐G_12_D mice and the control mice and monitored their appearance changes in the skin. Both groups of mice were treated with wax‐induced depilation for better examination of skin appearance and thereafter intraperitoneally administered with CNO for 10 consecutive days at a dose of 10 mg/kg/day (Figure 1C). After 10 days of CNO treatment, K14‐G_12_D mice developed prominent dorsal wrinkling, which may result from alterations in the dermis, and showed no hair regrowth (Figure 1D) [21]. Control mice showed regrown hair in the back skin. When control mice underwent wax depilation on day 10 in the experiment involving wax depilation on both day 0 and day 10, their skin exhibited only minimal wrinkling (Figure S2). When mice were further administered with CNO for 5 days, both genotypes of the mice developed hair growth (Figure S3). These findings suggest that K14‐G_12_D mice show impaired entry into the anagen phase. The epidermis was still significantly thicker in K14‐G_12_D mice than in control mice, even when wax depilation was performed after 10 days of CNO administration (Figure S4A,B). These results indicate that G_12_D activation in the epidermis induces skin morphological abnormalities such as wrinkles, possibly mimicking dermatitis [22]. Notably, careful inspection revealed no obvious erythema or scaling in either group (Figure 1D), indicating that G_12_D activation induces dermatitis‐like gross morphology without overt inflammatory signs.

We next performed histomorphological analysis of the back skin specimen. We found epidermal thickening in K14‐G_12_D mice (Figure 1E,F and Figure S4), particularly within the stratum granulosum and stratum spinosum. Despite these changes, the overall epidermal layers were preserved in both control and K14‐G_12_D mice, indicating preserved stratified architecture (Figure S5). The Masson's trichrome staining showed that the dermis was thicker in K14‐G_12_D mice compared with controls (Figure S6). It is possible that IL‐6 and TNFα, which are secreted by immune cells in the dermis, activate fibroblasts and induce dermal thickening [23, 24, 25, 26]. No morphological abnormalities were detected in hair follicles (Figures S5 and S6A,B). Taken together, activation of G_12_ signaling in the epidermis induces epidermal thickening without disrupting the layered structure, suggesting that G_12_ signaling promotes healthy epidermal thickening.

### 3.2 Gene Expression Changes Induced by G_12_ Signaling Activation in the Epidermis

**FIGURE 2:**
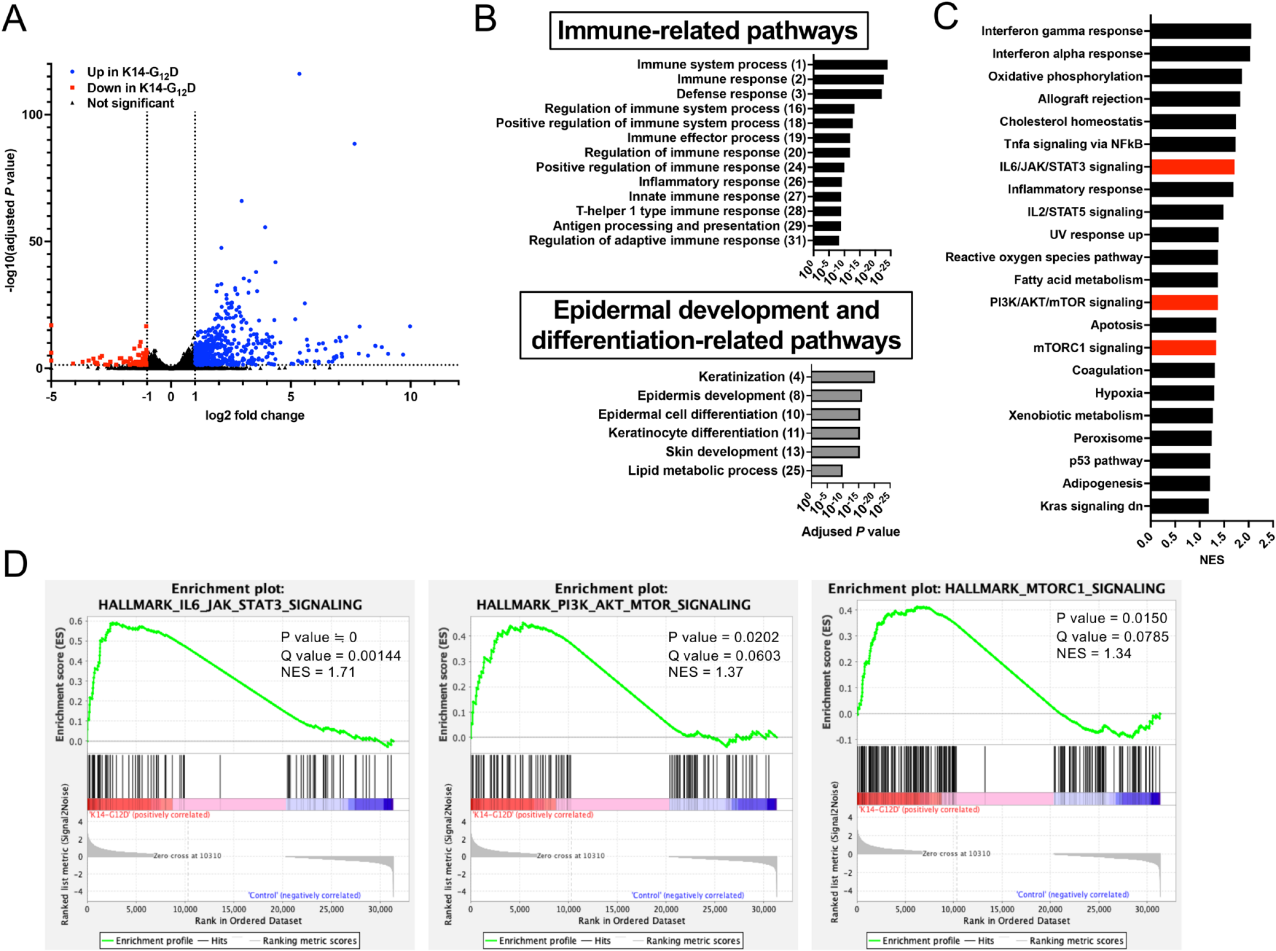
G_12_D activation induces expression of genes involved in keratinocyte differentiation, proliferation, and inflammation. (A) The volcano plot displays 524 entities of up‐regulated genes and 75 entities of down‐regulated genes with a 2‐fold cutoff and statistical significance (*p* < 0.05) in K14‐G_12_D mice (compared with control mice). (B) Enriched GO terms for genes with altered expression induced by G_12_ signaling activation. GO analysis was based on g:Profiler, and GO terms with *p‐*values below 1 × 10^−9^ are shown; among them, immune‐related and keratinocyte‐related terms were selected and presented. The numbers in the parentheses of the labels indicate the ranking of each term among the 224 significantly altered terms. (C) Gene set enrichment analysis (GSEA) of RNA‐seq data from control and K14‐G_12_D epidermis. Shown are the top significantly enriched Hallmark pathways ranked by normalized enrichment score (NES). Positive NES values indicate enrichment in K14‐G_12_D epidermis, whereas negative NES values indicate enrichment in control epidermis. Statistical significance was assessed using the false discovery rate (FDR). (D) GSEA plot showing significant enrichment of the Hallmark IL6–JAK–STAT3 signaling pathway, PI3K–AKT–mTOR signaling pathway, and mTORC1 signaling pathway in K14‐G_12_D mice compared to control mice.

To elucidate the mechanisms and signaling pathways responsible for the observed phenotypes, we performed RNA‐seq of whole skin from K14‐G_12_D and control mice. RNA was extracted from the whole dorsal skin. The volcano plot showed that, compared with control mice, 524 genes were upregulated, and 75 genes were downregulated in K14‐G_12_D mice, using cutoffs of adjusted *p* < 0.05 and absolute fold change > 2 (Figure 2A). While the expression of alarmin genes that function as early signals of innate immunity, such as *Il36g, Il18*, and *Tslp*, was increased in K14‐G_12_D mice, genes associated with Th2‐ or Th17‐mediated adaptive immune responses induced by alarmins were not upregulated. In contrast, the expression of genes associated with keratinocyte differentiation and enhanced epidermal function, such as *Loricrin*, *Krt10*, and *Hrnr*, was increased in K14‐G_12_D mice. These findings suggest that K14‐G_12_D mice activate innate immunity and enhance epidermal barrier function without inducing adaptive immune responses. Gene ontology (GO) analysis identified 224 biological pathways that were statistically (*p* < 0.05) significantly altered in response to activation of G_12_ signaling in the epidermis (Table S3). Of the 32 GO‐term pathways with an adjusted *p*‐value of less than 10^−9^ (Figure 2B), 6 pathways were related to keratinocyte differentiation or skin development, whereas 13 pathways were related to the immune system. In addition, gene set enrichment analysis (GSEA) showed that the IL‐6–JAK–STAT3, PI3K–AKT–mTOR, and mTORC1 signaling pathways were enriched in K14‐G_12_D mice (Figure 2C,D). Together, these results demonstrate that activation of G_12_ signaling in K14‐G_12_D mice enhances keratinocyte differentiation, immune responses, and mTORC1 signaling, suggesting that multiple pathways were altered by keratinocyte G_12_ activation.

### 3.3 G_12_ Signaling Activation in the Epidermis Induces Keratinocyte Proliferation and Differentiation

**FIGURE 3:**
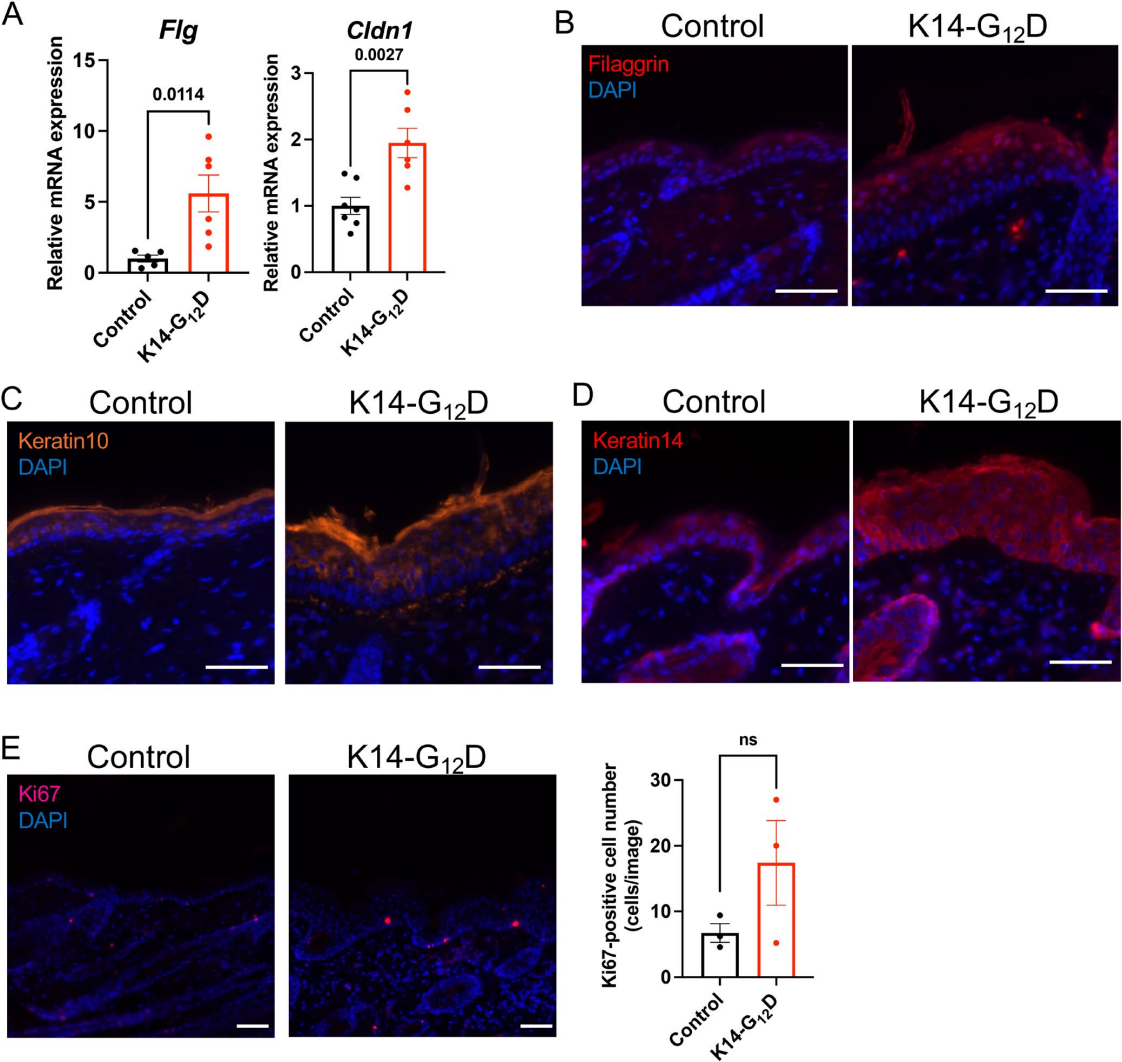
Epidermal activation of G_12_ signaling induces keratinocyte differentiation and proliferation. (A) Relative mRNA expression levels of *Flg* and *Cldn1* (*n* = 6–7 per group). Normalized fold changes for each gene are shown using *Gapdh* and *Hprt* as reference genes, respectively. (B–D) Representative images of immunostaining for filaggrin (B), keratin 10 (C), and keratin 14 (D) in dorsal skin sections. (E) Representative images of Ki67 immunostaining in dorsal skin sections and quantification of Ki67‐positive cell numbers (*n* = 3). Scale bars: 50 μm. Values represent mean ± SEM. Data were analyzed by the two‐tailed Student's *t*‐test.

Our RNA‐seq analysis revealed that activation of G_12_ signaling in the epidermis upregulated the keratinocyte differentiation pathway. Filaggrin is a terminal differentiation‐associated protein, whereas claudin‐1 is a tight junction component that contributes to epidermal barrier formation in differentiated keratinocytes [27, 28]. We therefore evaluated the expression of these markers. Skin samples collected after 10 days of CNO treatment were homogenized for mRNA extraction, and marker expression levels were quantified by RT‐qPCR (Figure 3A). In K14‐G_12_D mice, the mRNA levels of *Flg* and *Cldn1* were increased approximately five‐fold and two‐fold, respectively, compared with those in control mice (Figure 3A). We next examined filaggrin protein expression by immunostaining of dorsal skin sections from K14‐G_12_D and control mice. The filaggrin‐positive area was increased in K14‐G_12_D mice compared with control mice (Figure 3B). In addition, to further assess keratinocyte differentiation, we examined keratin 10 expression by immunostaining. The keratin 10‐positive layer was thicker in K14‐G_12_D mice than in control mice, indicating enhanced keratinocyte differentiation (Figure 3C). In addition, to examine whether the basal layer structure was maintained, we performed immunostaining of skin sections from K14‐G_12_D and control mice to assess keratin 14 expression. Keratin 14 expression was observed in the basal layer of both control and K14‐G_12_D mice (Figure 3D). Interestingly, in K14‐G_12_D mice, keratin 14 expression was also detected in layers above the basal layer, suggesting suprabasal persistence of keratin 14 expression during epidermal thickening in K14‐G_12_D mice.

Epidermal thickening can result from not only increased keratinocyte proliferation but also enhanced differentiation in K14‐G_12_D mice. To investigate whether keratinocyte proliferation increases in K14‐G_12_D mice, we performed immunostaining for Ki67, a proliferation marker. The number of Ki67‐positive cells tended to increase in K14‐G_12_D mice, indicating enhanced proliferative activity in the epidermis (Figure 3E). Taken together, these findings suggest that activation of G_12_ signaling in the epidermis promotes both keratinocyte differentiation and proliferation, leading to expansion of both the basal cell‐like region and the differentiated cell layers and contributing to enhanced epidermal barrier function.

### 3.4 Epidermal G_12_D Activation Enhances Skin Barrier Function

**FIGURE 4:**
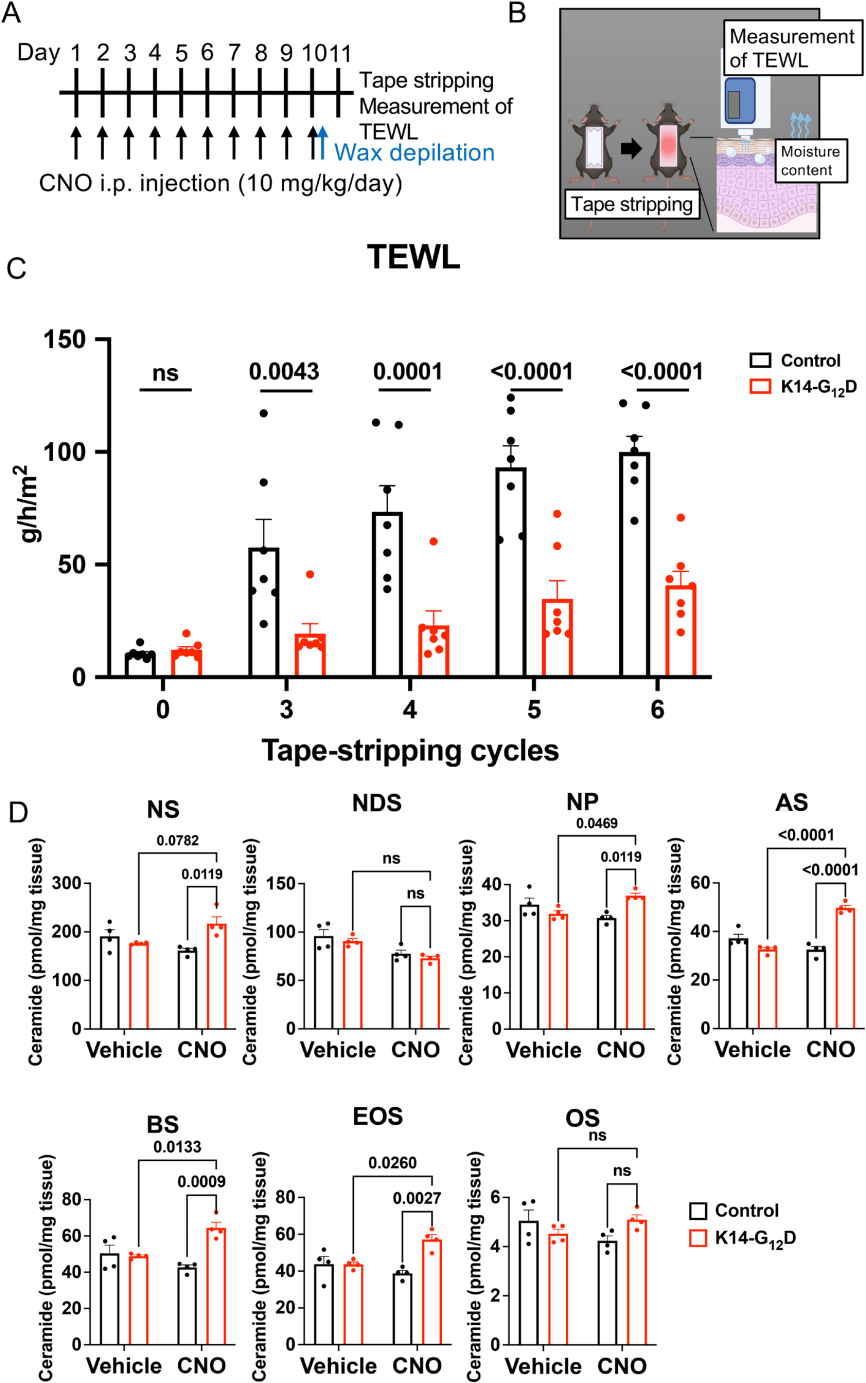
Epidermal G_12_D activation enhances epidermal barrier function. (A) Time course for CNO administration and wax depilation and measurement of transepidermal water loss (TEWL). Eight‐week‐old female K14‐G_12_D and control mice received daily i.p. injections of CNO (10 mg/kg/day) for 10 days, followed by wax depilation. (B) Schematic diagram of TEWL measurement. (C) TEWL score of K14‐G_12_D mice and control mice (*n* = 7 per group). (D) Amount of each ceramide class in mouse skin. Lipids were extracted from dorsal skin and subjected to LC/MS/MS analyses to quantify ceramides. Values are the sum of the ceramide species containing each FA chain length (nonhydroxy ceramides, α‐hydroxy ceramides, and BS: C14‐C36; *ω*‐hydroxy and EO ceramides: C26‐36). Values represent mean ± SEM. Data were analyzed by the two‐way ANOVA followed by the Sidak's post hoc tests (C) or the two‐tailed Student's *t*‐test (D).

Given that the K14‐G_12_D mice showed an apparently intact, thickened epidermis and increased filaggrin and claudin‐1 expression, we next assessed their epidermal barrier functions. We administered CNO at 10 mg/kg/day for 10 days. Mice were treated with wax depilation performed on day 10 instead of wax depilation on day 0, which allowed us to assess transepidermal water loss (TEWL) measurements under hair depilated conditions (Figure 4A). On the following day, we measured TEWL before and after tape stripping to physically disrupt the epidermal barrier (Figure 4A,B). Prior to tape stripping, baseline TEWL values were comparable between K14‐G_12_D mice and control mice (Figure 4C). Following repeated tape stripping, control mice showed increased TEWL, reaching 89 g/h/m^2^ after the sixth round of tape stripping. K14‐G_12_D mice showed a gradual TEWL response, reaching 28 g/h/m^2^ after the sixth round. While control mice showed a marked increase in TEWL during the third round of tape stripping, K14‐G_12_D mice exhibited almost no change in TEWL at this stage, demonstrating a pronounced resistance to low‐level barrier disruption. To determine whether the reduced TEWL observed in K14‐G_12_D mice was attributable to an increased amount of stratum corneum–associated proteins or to an increased number of stratum corneum layers, we quantified the total protein extracted from each tape strip. No significant difference in protein content was detected between control mice and K14‐G_12_D mice (Figure S7). Tape stripping was performed under standardized conditions, including comparable pressure, contact time, and stripping procedure, to minimize technical variation. Because quantification of protein recovered from tape strips is widely used as an estimate of the amount of stratum corneum removed, we compared the protein content recovered per strip between the two groups; the comparable content suggests that the protein content of the superficial stratum corneum removed under these conditions was not markedly increased in K14‐G_12_D mice. The attenuated TEWL response in K14‐G_12_D mice is therefore unlikely to be explained solely by increased protein content in individual superficial stratum corneum removed by each strip. Increased stratum corneum layering and/or qualitative changes in barrier components may instead contribute to this phenotype. These results suggest that activation of epidermal G_12_ signaling enhances epidermal barrier function.

We next examined ceramide levels, which play a critical role in water retention within the epidermal barrier. K14‐G_12_D and control mice were treated with CNO for 10 days. On the day after the final administration, dorsal skin was shaved and collected. Lipids were extracted from the collected skin and analyzed by LC–MS/MS to quantify the top 100 most abundant ceramide species in the mouse stratum corneum. As a result, CNO‐treated K14‐G_12_D mice showed significantly increased levels of ceramide *N*‐(non‐hydroxyacyl)‐sphingosine (NS), *N*‐(non‐hydroxyacyl)‐phytosphingosine (NP), *N*‐(*α*‐hydroxyacyl)‐sphingosine (AS), *N*‐(*β*‐hydroxyacyl)‐sphingosine (BS), and *N*‐(*ω*‐hydroxyacyl)‐eicosasphingenine (EOS) compared with CNO‐treated control mice (Figure 4D). Furthermore, NP, AS, BS, and EOS levels were significantly increased in CNO‐treated K14‐G_12_D mice relative to saline‐treated K14‐G_12_D mice (Figure 4D). Among these ceramide species, NS (16:0), NP (22:0), AS (16:0), BS (24:0 and 26:0), and EOS (32:0) showed particularly robust increases (Figure S8). Notably, EOS has been reported to play a key role in epidermal barrier function, with increased EOS enhancing barrier integrity and promoting water retention [29]. These findings suggest that activation of G_12_D signaling in the epidermis increases ceramide content and enhances epidermal barrier function, particularly its water‐holding capacity.

### 3.5 G_12_D Activation in the Epidermis Induces Weak Inflammation

**FIGURE 5:**
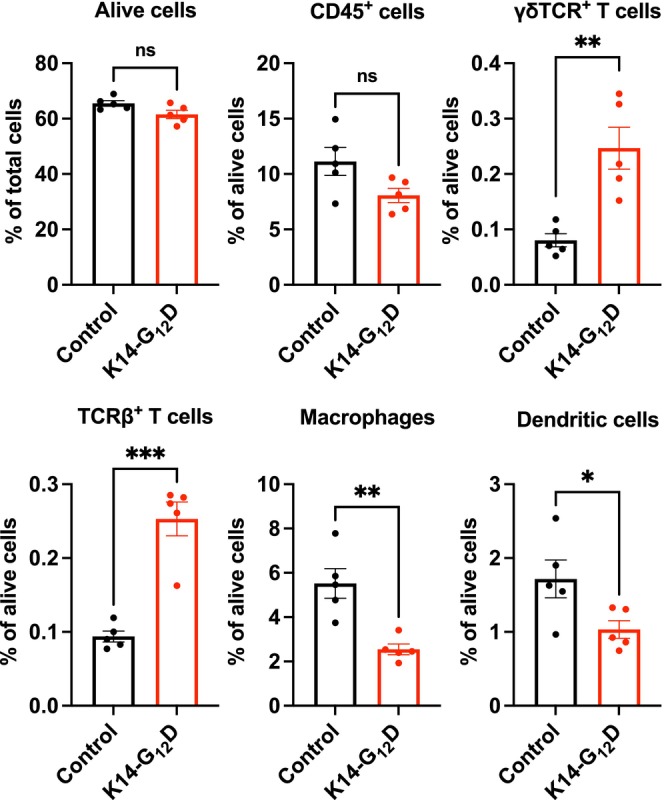
G_12_ signaling activation in the epidermis induced changes in immune cell infiltration. Flow cytometric analysis was performed using dorsal skin cells isolated from control and K14‐G_12_D mice. The proportions of living cells, CD45^+^ cells, γδTCR^+^ T cells, TCRβ^+^ T cells, macrophages, and dendritic cells are shown as percentages of total cells. Each dot represents one mouse. Values represent mean ± SEM. Data were analyzed by the two‐tailed Student's *t*‐test.

Our RNA‐seq analysis indicated upregulated inflammatory pathways in K14‐G_12_D mice (Figure 2). To investigate changes in immune cell infiltration into skin, we performed flow cytometric analysis. K14‐G_12_D and control mice were administered CNO for 10 days starting on the day after wax depilation. Thereafter, we enzymatically dissociated dorsal skin into single cells and analyzed them by flow cytometry (Figure S9). We found that the proportion of CD45^+^ cells was comparable between K14‐G_12_D mice and control mice (Figure 5). The proportions of macrophages and dendritic cells were decreased in K14‐G_12_D mice. In contrast, the proportions of γδT cells and TCRβ^+^ T cells were significantly increased in K14‐G_12_D mice. These results show that activation of G_12_ signaling in the epidermis alters the composition of immune cells in the whole skin.

To investigate whether K14‐G_12_D mice develop inflammation, we assessed the mRNA expression of inflammatory markers. Since γδT cells, which increased in the K14‐G_12_D mouse skin (Figure 5), are known to accumulate in psoriatic lesions [30], and IL‐23 is involved in the initiation and exacerbation of psoriasis [30], we measured IL‐23 in addition to general inflammatory cytokines including IL‐6 and TNFα. Skin samples collected on day 10 of CNO treatment were homogenized for mRNA extraction, and marker expression levels were analyzed by RT‐qPCR. The mRNA expression levels of *Il6* and *Tnfa* were significantly higher in K14‐G_12_D mice than in control mice (Figure S10). However, *Il6* and *Tnfa* typically show a 10‐fold or greater increase under inflammatory conditions; therefore, the modest elevations observed in K14‐G_12_D mice are unlikely to reflect overt inflammation. In contrast, the mRNA level of *Il23* was also significantly higher in K14‐G_12_D mice compared with controls (Figure S10). K14‐G_12_D mice showed epidermal thickening, increased γδT‐cell populations, and elevated *Il23* mRNA levels, all of which are indicative of psoriasis‐like features [31]. Taken together, these results indicate that G_12_ signaling activation does not cause marked inflammation, but it partially shifts the epidermal cytokine environment toward a psoriasis‐associated pattern.

### 3.6 TYK2 Partially Contributes to G_12_D‐Induced Epidermal Thickening

**FIGURE 6:**
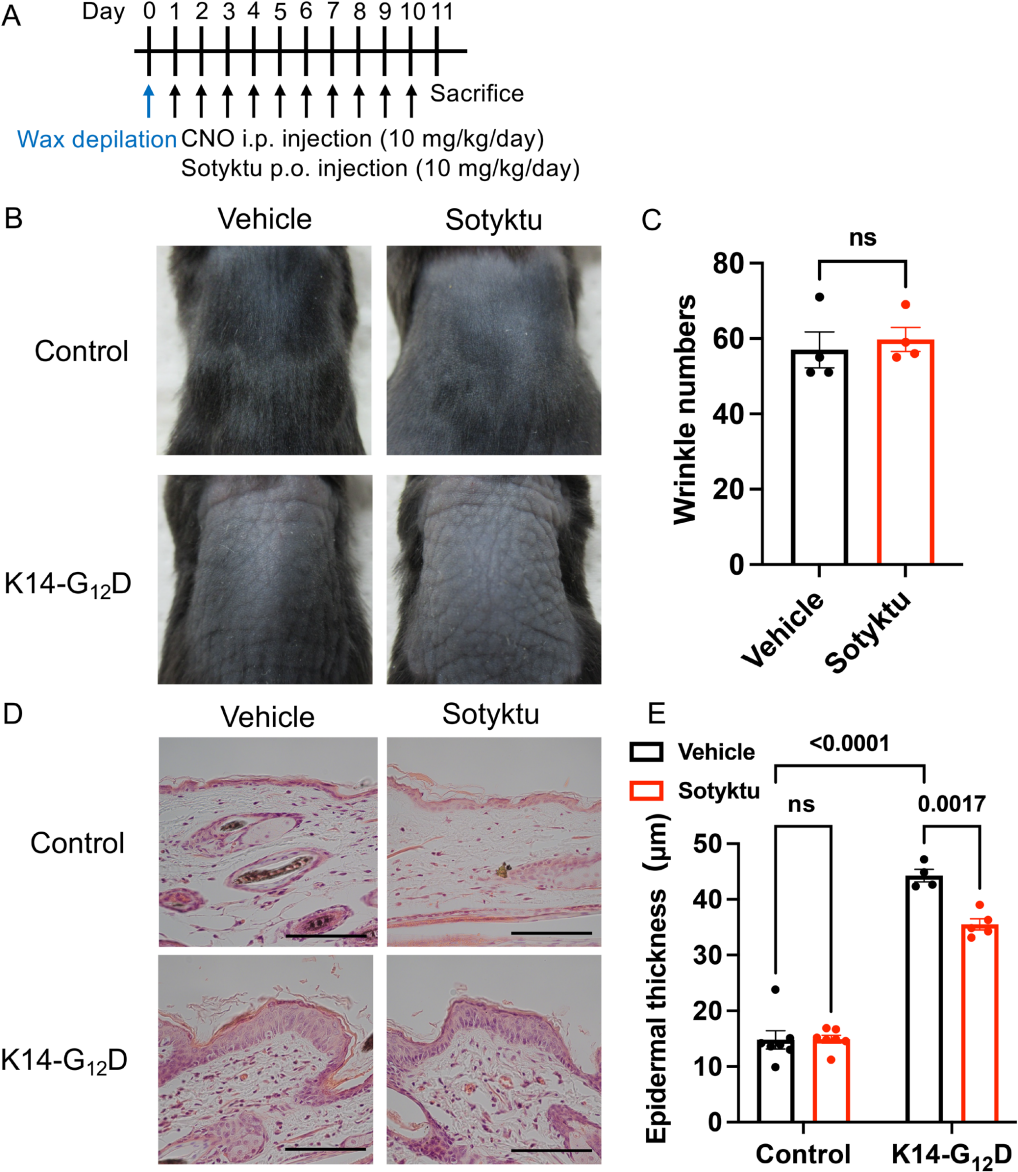
TYK2 is involved in the epidermal thickening of K14‐G_12_D mice. (A) Protocol for CNO administration and Sotyktu administration. Eight‐week‐old female K14‐G_12_D and control mice were injected i.p. CNO (10 mg/kg/day) and p.o. Sotyktu (10 mg/kg/day) after wax depilation. (B) Representative appearance of K14‐G_12_D and control mice with or without Sotyktu. (C) Quantification of wrinkle number of K14‐G_12_D mice shown in (B) (*n* = 4 per group). (D) Representative HE staining of dorsal skin specimens. Scale bars: 100 μm. (E) Quantification of epidermal thickness shown in (D) (*n* = 4–7 per group). Values represent mean ± SEM. Data were analyzed by the two‐tailed Student's *t*‐test (C) or the two‐way ANOVA followed by the Sidak's post hoc tests (E).

In psoriasis, IL‐23 produced by dendritic cells binds to cytokine receptors expressed on helper T cells (Th17), leading to the association of TYK2 and JAK2 [32]. This interaction activates the downstream signaling molecule STAT3, which in turn induces inflammation and keratinocyte proliferation [33, 34]. To date, therapeutic drug development for psoriasis has focused on targets such as the IL‐23/IL‐17 axis and TNFα [33]. More recently, TYK2 has emerged as an important therapeutic target, and the TYK2 inhibitor deucravacitinib has been approved by the FDA for the treatment of psoriasis [35]. As shown above, psoriasis‐associated cytokine changes were observed in K14‐G_12_D mice, and RNA‐seq analysis revealed that the IL‐6–JAK–STAT3 pathway is enhanced in K14‐G_12_D mice, suggesting that TYK2 may be involved in the epidermal thickening seen in this model. By using Sotyktu (deucravacitinib), a TYK2 inhibitor, we assessed the role of TYK2 in G_12_D‐induced epidermal thickening. We prepared four groups of mice: two mouse lines (K14‐G_12_D and control) with two treatment conditions (Sotyktu and vehicle). For all four groups, we administered CNO intraperitoneally and Sotyktu or vehicle orally for 10 days beginning the day after hair depilation (Figure 6A). In the control mouse groups, the back skin exhibited only mild wrinkling regardless of the Sotyktu treatment (Figure 6B). In the vehicle‐treated K14‐G_12_D mice, the back skin developed pronounced wrinkling. Skin appearance was comparable between vehicle‐ and Sotyktu‐treated K14‐G_12_D mice (Figure 6B,C). We then analyzed epidermal thickening by the histology of the skin specimens of these mice. Consistent with previous experiments, K14‐G_12_D mice showed increased epidermal thickness compared with control mice in the vehicle condition (Figure 6D,E and Figure S11). When compared between the vehicle and the Sotyktu groups in the K14‐G_12_D mice, Sotyktu treatment partially reduced epidermal thickness. Sotyktu treatment had little effect on epidermal thickness between treatment groups within the control genotype.

To investigate whether TYK2 inhibition affects immune cell infiltration, we assessed the number of CD3^+^ T cells in the skin. Dorsal skin sections from K14‐G_12_D and control mice were stained with an anti‐CD3 antibody, and the number of CD3^+^ T cells was quantified. The number of CD3^+^ T cells was comparable between both Sotyktu‐treated and vehicle‐treated groups in both K14‐G_12_D and control mice (Figure S12A,B). These results showed that TYK2 did not affect CD3^+^ T cells in the skin. Taken together, these findings indicate that increased epidermal thickness in K14‐G_12_D mice is partly mediated through the TYK2–STAT3 axis.

### 3.7 G_12_ Signaling Induced Epidermal Thickening Is Mediated by mTORC1 Signaling

**FIGURE 7:**
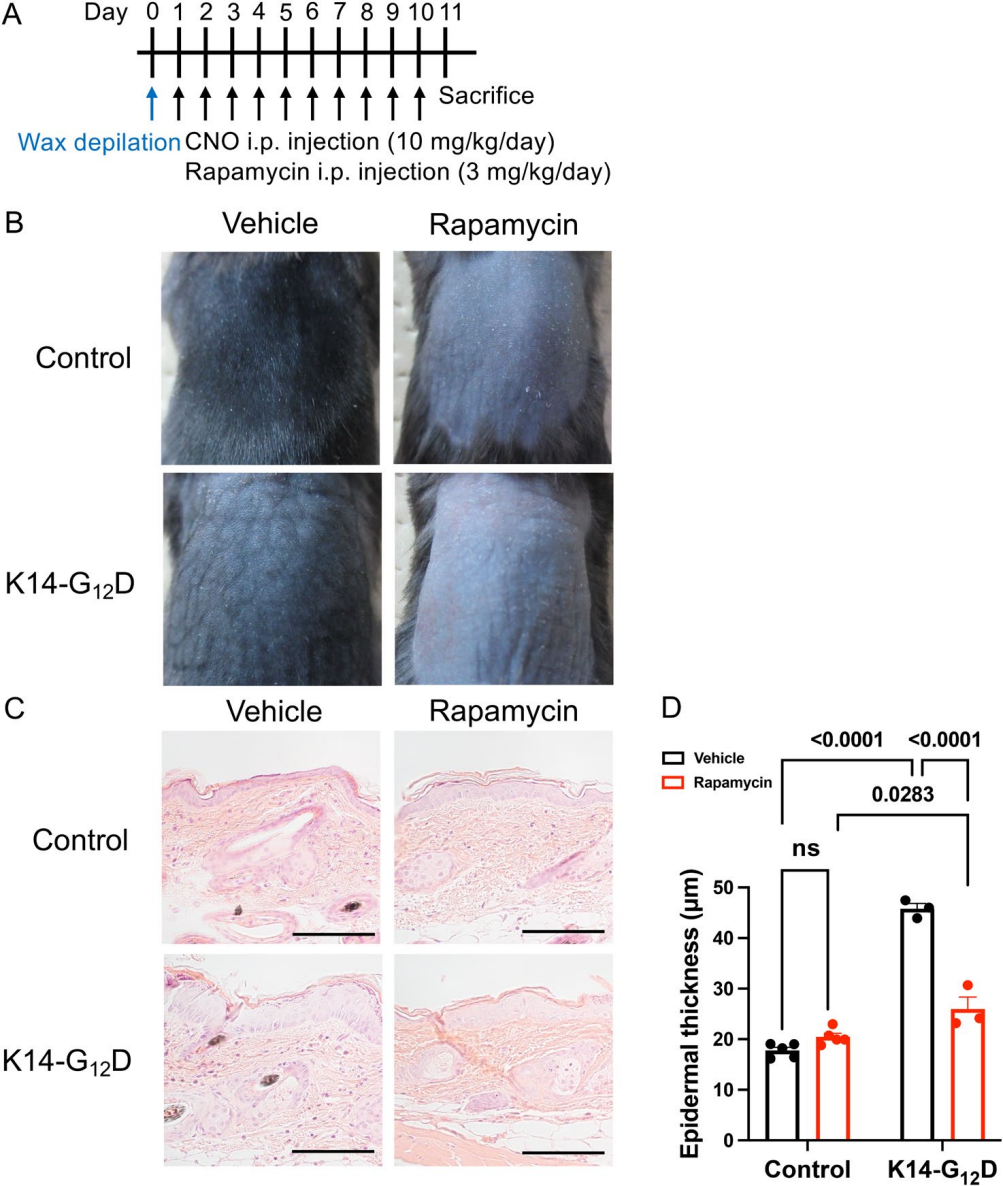
G_12_‐induced epidermal thickening is mediated by mTORC1 signaling. (A) Protocol for CNO administration and rapamycin administration. Eight‐week‐old female K14‐G_12_D and control mice were injected i.p. CNO (10 mg/kg/day) and rapamycin (3 mg/kg/day) after wax depilation. (B) Representative appearance of K14‐G_12_D and control mice with or without rapamycin. (C) Representative HE staining of dorsal skin specimens. Scale bars: 100 μm. (D) Quantification of epidermal thickness shown in (C) (*n* = 3–5 per group). Values represent mean ± SEM. Data were analyzed by the two‐way ANOVA followed by the Sidak's post hoc tests.

Our RNA‐seq analysis revealed upregulation of the mTORC1 signaling pathway in K14‐G_12_D mice. Because mTORC1 signaling is known to be involved in cell proliferation [36], we next investigated whether mTORC1 signaling is responsible for G_12_D‐induced epidermal thickening. K14‐G_12_D and control mice were intraperitoneally administered CNO (10 mg/kg/day) together with either rapamycin (3 mg/kg/day) or vehicle for 10 days starting on the next day of wax depilation (Figure 7A). In control mice, the dorsal skin exhibited only mild wrinkling regardless of rapamycin treatment (Figure 7B). In vehicle‐treated K14‐G_12_D mice, the dorsal skin showed pronounced wrinkling, whereas wrinkling was attenuated in rapamycin‐treated K14‐G_12_D mice. Hair regrowth was delayed in rapamycin‐treated groups, consistent with the known inhibitory effect of rapamycin on stem cell proliferation. To evaluate epidermal thickening, we measured the epidermal thickness of dorsal skin sections. Vehicle‐treated K14‐G_12_D mice showed increased epidermal thickness compared with control mice (Figure 7C,D). In contrast, rapamycin‐treated K14‐G_12_D mice showed a statistically significant reduction in epidermal thickness compared with vehicle‐treated K14‐G_12_D mice. Rapamycin did not affect epidermal thickness in control mice. These results indicate that mTORC1 signaling contributes to G_12_ signaling‐induced epidermal thickening and wrinkle formation.

### 3.8 Multiple G_12_ ‐Coupled GPCRs Are Expressed in the Epidermis

**FIGURE 8:**
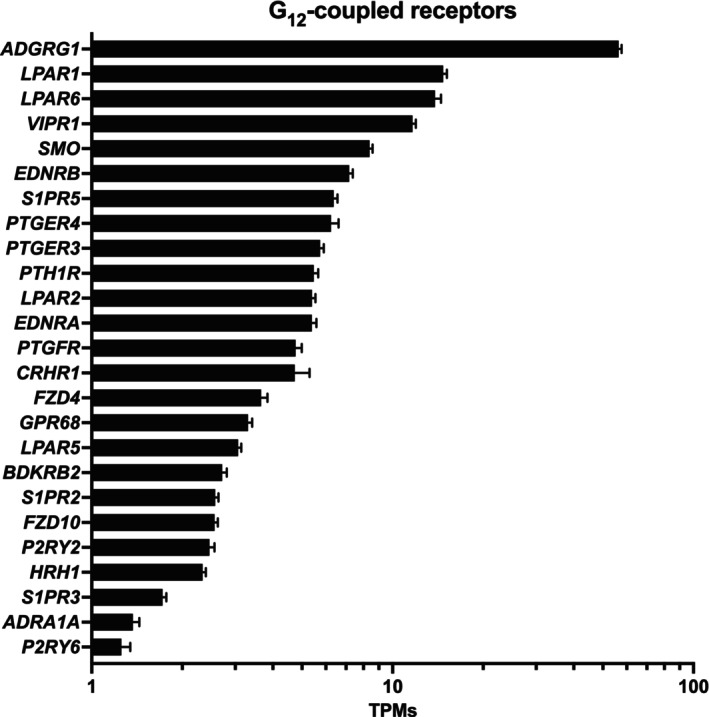
G_12_‐coupled GPCRs expressed in normal human skin. TPM values for genes encoding G_12_‐coupled GPCRs in healthy humans are shown (*n* = 82). Values represent mean ± SEM.

To investigate the presence of endogenous G_12_‐coupled GPCRs in the epidermis, we analyzed their expression using publicly available comprehensive mRNA datasets (GSE: 54456). We first compiled a list of GPCRs expressed in human skin based on previously reported RNA‐sequencing data. Next, G_12_‐coupled GPCRs were identified using the Guide‐to‐Pharmacology Database and two published studies [12, 37, 38]. GPCRs with expression levels of TPM > 1 in human skin were selected for further analysis (Figure 8). We then determined which of these GPCRs couple to G_12_ by cross‐referencing published reports. This analysis identified GPCRs such as *Lpar1*, *Lpar5*, and *S1pr2*, which have been reported to enhance epidermal barrier function, as well as GPCRs such as *Lpar2*, whose roles in the skin remain poorly defined [10, 39]. These findings suggest that multiple G_12_‐coupled GPCRs are present in the epidermis and may collectively contribute to the regulation of epidermal barrier function.

## 4 Discussion

In this study, we identify epidermal G_12_ signaling as a driver of barrier‐supportive epidermal expansion in vivo. Using keratinocyte‐specific activation of G_12_ signaling (K14‐G_12_D), we show that G_12_ activation induces robust epidermal thickening while concurrently enhancing epidermal barrier function. This combination—epidermal thickening with preserved differentiation and improved barrier performance—supports a “non‐pathological” thickening phenotype and expands our understanding of how GPCR pathways can tune epidermal homeostasis.

The epidermal thickening and enhancement of barrier function observed upon G_12_D activation are presumed to be mediated through G_12_‐dependent signaling pathways. Prior work indicates that GPCR signaling can promote or impair epidermal growth and barrier repair depending on the engaged G‐protein pathway [7, 8, 9, 40, 41]. Activation of the G_s_‐coupled GPCR EP2 induces excessive keratinocyte proliferation, whereas activation of the G_i_‐coupled GPCRs CB1 and β_2_AR impairs epidermal barrier function [7, 8, 40]. In addition, activation of a G_i_‐coupled DREADD also promotes excessive keratinocyte proliferation [41]. Furthermore, activation of the G_q_‐coupled H1R suppresses keratinocyte differentiation, suggesting compromised epidermal barrier function [9]. Collectively, these findings indicate that the phenotype observed upon G_12_D activation differs from those resulting from G_s_‐, G_i_‐, or G_q_‐mediated signaling. Consistent with this, an in vitro keratinocyte study showed that activation of LPAR1 and LPAR5, GPCRs known to couple to G_12_, enhances keratinocyte differentiation, increases FLG expression, and improves epidermal barrier function—findings consistent with our observations [10]. In line with our findings, previous studies have reported that loss of S1PR2, a GPCR capable of coupling to G_12_, decreases expression of tight junction–associated proteins and filaggrin‐2 [39]. Additionally, another G_13_‐coupled receptor ADGRL2 has been shown to promote keratinocyte differentiation [42]. Thus, our in vivo results are consistent with a model in which G_12_‐linked programs promote differentiation‐compatible epidermal expansion and barrier reinforcement.

A plausible mechanistic route involves the canonical G_12_–RhoA–ROCK axis and its downstream signaling factors, SRF and YAP/TAZ. SRF has been implicated in keratinocyte differentiation and induction of barrier‐associated genes [43], whereas YAP/TAZ is known to promote keratinocyte proliferation [44]. Consistent with this framework, filaggrin expression is induced through the LPA–LPAR1/5–RhoA–ROCK–SRF signaling axis, and these receptors are likewise known to signal through G_12_ [10]. In K14‐G_12_D mice, filaggrin protein expression was increased, suggesting possible involvement of the G_12_–RhoA–ROCK–SRF pathway in this phenotype. In addition, G_12_‐coupled receptors LPAR1 and LPAR3 have been reported to induce YAP activation through the G_12_–RhoA–actin cytoskeleton–Lats1 pathway [45]. This finding suggests that this pathway also contributes to epidermal thickening, particularly to the enhanced keratinocyte proliferation observed in this study. In the present study, epidermis‐specific activation of G_12_ signaling increased keratinocyte differentiation and proliferation, raising the possibility that SRF and YAP/TAZ contribute to the expansion of differentiated layers and the basal cell‐like compartment, respectively.

Epidermal activation of G_12_ signaling may share some features with keratinocyte responses induced during wound repair or barrier perturbation. In the present study, alarmin‐related genes, including *Tslp*, *Il18*, and *Il36*, were upregulated in K14‐G_12_D mice. Alarmins function as damage‐associated molecular patterns (DAMPs) released in response to tissue injury or cellular stress, and they initiate innate immune and tissue responses during wound healing and barrier disruption [46, 47]. Because alarmins also induce or amplify T cell responses through dendritic cells and innate immune cells, the upregulation of alarmin‐related genes could potentially lead to inflammatory T cell responses [46]. Although γδT cells and TCRβ^+^ T cells were increased in K14‐G_12_D mice, inflammatory T cell effector cytokines such as *Il17* were not markedly induced, suggesting that typical adaptive immune‐driven inflammation was not strongly elicited. Thus, the upregulation of alarmin‐related genes observed in K14‐G_12_D mice may reflect a keratinocyte‐intrinsic stress or tissue repair‐like response that partially overlaps with early wound or barrier repair responses, rather than an overt inflammatory skin lesion.

Our pharmacological analyses indicated that TYK2‐dependent signaling and mTORC1 activity contribute to G_12_‐induced epidermal thickening. TYK2 is a member of the JAK family that mediates signaling downstream of cytokine receptors, including those for IL‐12, IL‐23, and type I interferons [48]. In epithelial cells, IL‐22 can activate JAK1/TYK2–STAT3 signaling and regulate proliferative and tissue‐remodeling responses [48]. Functional crosstalk between G_12_ and JAK–STAT signaling has been reported in selected cellular contexts [49]. In addition, JAK1 activation induces actomyosin contractility through the RhoA–ROCK pathway [50]. Thus, the partial suppression of G_12_D‐induced epidermal thickening by TYK2 inhibition suggests that TYK2‐dependent cytokine signaling contributes to the phenotype. mTORC1 plays an important role in basal keratinocyte proliferation, cellular growth, and maintenance of the multilayered epidermis [51]. Previous studies have also linked G_12_ signaling to mTORC1 activation in other cellular contexts. In skeletal muscle, activation of the G_12_‐coupled receptor GPR56 induces IGF1 expression and subsequently activates mTORC1 signaling [52]. In addition, recent evidence suggests that Gα_12_ supports Akt–mTORC1–p70S6K signaling and protein synthesis in myotubes [53]. Although the molecular connection between G_12_ and mTORC1 in keratinocytes remains unclear, the inhibitory effect of rapamycin in our model suggests that mTORC1‐dependent growth programs contribute substantially to G_12_D‐induced epidermal thickening. Notably, mTORC1 inhibition produced a stronger suppressive effect than TYK2 inhibition. This difference may reflect the position of mTORC1 as a central integrator of multiple growth factor, nutrient, metabolic, and cytokine‐derived signals that converge on protein synthesis, cellular growth, and proliferation. In contrast, TYK2 inhibition affects a more restricted subset of cytokine receptor pathways and may therefore suppress only the cytokine‐dependent component of the phenotype. The stronger effect of rapamycin is therefore consistent with a broader contribution of mTORC1‐dependent programs to G_12_D‐induced epidermal remodeling.

G_12_‐mediated enhancement of epidermal barrier function has potential therapeutic implications. Epidermal barrier integrity is closely linked to inflammatory skin diseases such as psoriasis and atopic dermatitis [4, 54], and impaired barrier function contributes to both disease onset and exacerbation [4, 5]. Expression of key barrier components—including FLG, CLDN1, and ceramides—is reduced in patients with these conditions [55, 56, 57]. Because many current therapies improve barrier function indirectly via immunosuppression [58, 59], strategies that directly reinforce epidermal barrier programs remain an important opportunity. Supporting this concept, tapinarof, an AHR agonist, ameliorates atopic dermatitis and psoriasis by increasing FLG expression in keratinocytes [60]. In this study, activation of epidermal G_12_ signaling increased expression of FLG, CLDN1, and ceramides and enhanced barrier function. Several G_12_‐coupled GPCRs are expressed in skin, and receptors such as LPAR1/5 and S1PR2 have been reported to promote epidermal barrier function [10, 39]. Collectively, our findings motivate future efforts to test whether pharmacologic modulation of endogenous epidermal G_12_‐coupled GPCRs can strengthen barrier function in disease‐relevant contexts.

Limitations of the current study include that our analyses were performed on the general epidermal cell population, and that the causal relationship between epidermal thickening and enhanced barrier function remains unresolved. Because G_12_D expression was driven by the *Krt14* promoter, G_12_ signaling was broadly activated across basal keratinocytes; identifying the most critical epidermal cell populations will require more refined genetic approaches. In addition, while epidermal thickening and barrier enhancement co‐occur, the causal relationship between these outputs remains to be defined. We observed increased expression of claudin genes, suggesting that intercellular adhesion is preserved even when the epidermis becomes thickened. However, because barrier function is multifactorial, it is difficult to determine whether increased cell layers with preserved adhesion are sufficient to account for the improved barrier [61]. Moreover, disruption of cell adhesion can also result in epidermal thickening, making it challenging to fully separate the effects of adhesion from thickening [62]. Future studies that decouple proliferation/stratification from barrier remodeling will be important to establish causality.

## Author Contributions

Nozomi Kamakura designed and conducted experiments, prepared figures, results, and wrote part of the manuscript; Natsumi Hirai designed and conducted experiments and prepared figures, results; Kaito Arai designed experiments and wrote part of the manuscript; Yaxin Du, Toshiaki Kogame, and Yusuke Ohno conducted experiments; Akio Kihara and Kenji Kabashima supervised experiments; Asuka Inoue designed the study and wrote the manuscript.

## Funding

This work was supported by the Japan Society for the Promotion of Science (JSPS; KAKENHI grants JP21H04791, JP24K21281, and JP25H01016), the Japan Agency for Medical Research and Development (AMED; grants JP22ama121038 and JP22zf0127007), the Japan Science and Technology Agency (JST; grants JPMJFR215T and JPMJMS2023), and The Uehara Memorial Foundation.

## Conflicts of Interest

The authors declare no conflicts of interest.

## Supporting information


**Figure S1:** G_12_D expression in K14‐G_12_D and control mice.
**Figure S2:** Effect of G_12_D activation on epidermis.
**Figure S3:** Effect of G_12_D activation on hair cycle.
**Figure S4:** Effect of G_12_D activation in the absence of wax depilation.
**Figure S5:** Low magnification images of HE staining of skin sections.
**Figure S6:** Effect of G_12_D activation on dermis.
**Figure S7:** Protein amount in stratum corneum of mouse upon chemogenetic G_12_D activation.
**Figure S8:** Ceramide profile in mouse skin upon chemogenetic G_12_D activation.
**Figure S9:** Gating strategy for flow cytometric analysis of immune cell populations in dorsal skin.
**Figure S10:** Effect of G_12_D activation on cytokine expression.
**Figure S11:** Low magnification images of HE staining of skin sections.
**Figure S12:** Immunostaining of dorsal skin specimens with anti‐CD3 antibody.
**Table S1:** RT‐qPCR primers used in this study.
**Table S2:** MRM settings for LC/MS/MS analysis of ceramide species.
**Table S3:** Upregulated GO terms in K14‐G_12_D mice.

## Data Availability

All of the data supporting the findings of the present study are available within the paper.
