# Supplementary material for: Chemogenetic Activation of G12 Signaling Thickens the Epidermis With Enhanced Barrier Function
Source: FASEB Bioadv. 2026 Jul 31;8(8):e70138. doi: 10.1096/fba.2026-00042 (PMC13426331; doi:10.1096/fba.2026-00042)
Supplement: Supplementary file 1 — Figure S1: G12D expression in K14‐G12D and control mice. Figure S2: Effect of G12D activation on epidermis. Figure S3: Effect of G12D activation on hair cycle. Figure S4: Effect of G12D activation in the absence of wax depilation. Figure S5: Low magnification images of HE staining of skin sections. Figure S6: Effect of G12D activation on dermis. Figure S7: Protein amount in stratum corneum of mouse upon chemogenetic G12D activation. Figure S8: Ceramide profile in mouse skin upon chemogenetic G12D activation. Figure S9: Gating strategy for flow cytometric analysis of immune cell populations in dorsal skin. Figure S10: Effect of G12D activation on cytokine expression. Figure S11: Low magnification images of HE staining of skin sections. Figure S12: Immunostaining of dorsal skin specimens with anti‐CD3 antibody. Table S1: RT‐qPCR primers used in this study. Table S2: MRM settings for LC/MS/MS analysis of ceramide species. Table S3: Upregulated GO terms in K14‐G12D mice. [file FBA2-8-e70138-s001.pdf]

Chemogenetic activation of G_{12} signaling thickens the epidermis with enhanced barrier function

Nozomi Kamakura[1,2,#], Natsumi Hirai[2,#], Kaito Arai[1,2,#], Yaxin Du[3], Toshiaki Kogame[3], Yusuke Ohno[4,5], Akio Kihara[4], Kenji Kabashima[3], Asuka Inoue[1,2,*]

[1]Graduate School of Pharmaceutical Sciences, Kyoto University, Kyoto, 606-8501 Japan

[2]Graduate School of Pharmaceutical Sciences, Tohoku University, Sendai, 980-8578 Japan

[3]Department of Dermatology, Graduate School of Medicine, Kyoto University, Kyoto, 606-8507 Japan

[4]Faculty of Pharmaceutical Sciences, Hokkaido University, Sapporo, 060-0808 Japan

[5]Faculty of Pharmacy, Juntendo University, Chiba, 279-0013 Japan

#These authors contributed equally

*corresponding author: aska@pharm.kyoto-u.ac.jp

Figure S1−S12

Table S1−S3

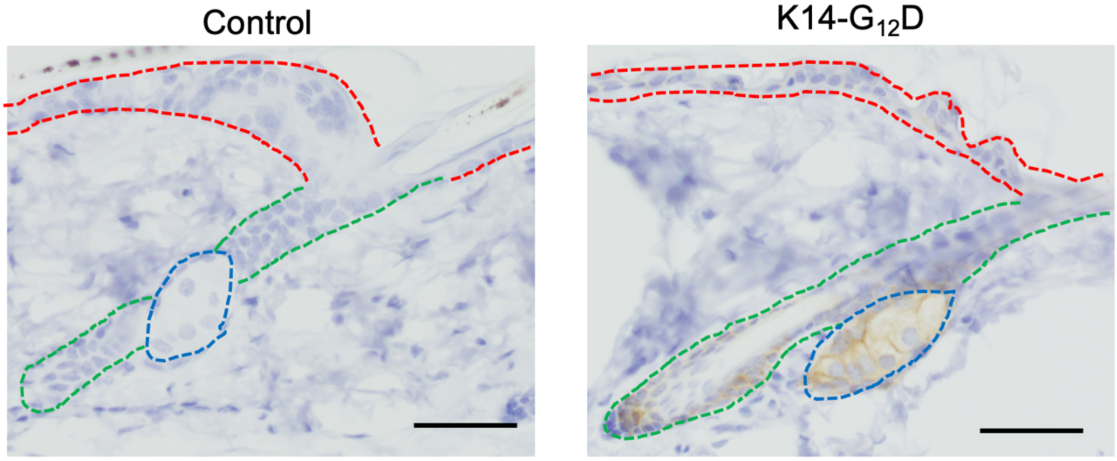

Figure S1. $G_{12}D$ expression in K14-$G_{12}D$ and control mice.

Representative immunostaining images of $G_{12}D$ expression in K14-$G_{12}D$ and control mice. Red: epidermis; green: hair follicle; blue: sebaceous gland. Scale bars: 20 µm.

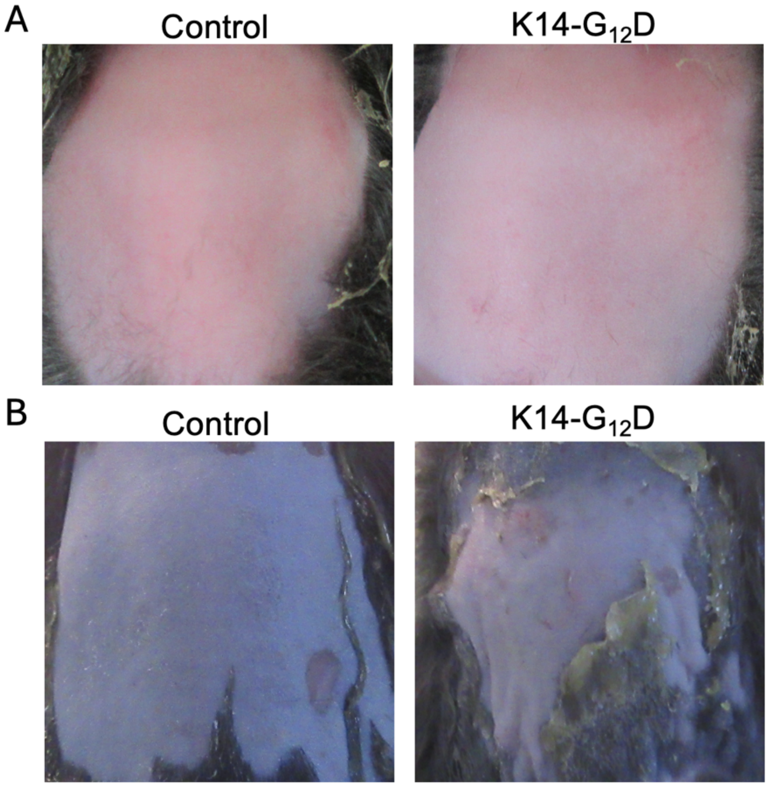

Figure S2. Effect of $G_{12}D$ activation on epidermis.

A, Representative images of dorsal skin depilated with wax, photographed before CNO administration.

B, Representative images of dorsal skin depilated with wax, then injected daily with CNO (10 mg/kg/day) for 10 days and depilated again with wax at the end of the treatment period (n = 5 per group).

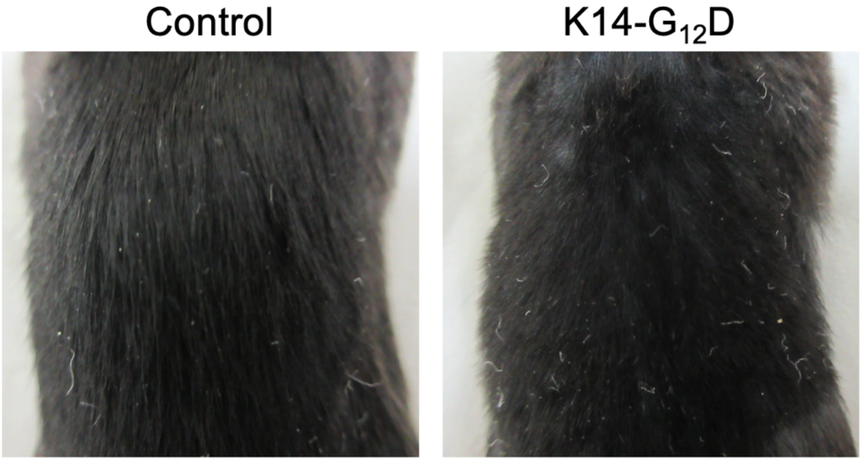

Figure S3. Effect of $G_{12}D$ activation on hair cycle.

Mice were daily injected daily with CNO (10 mg/kg/day) for 15 days (*n* = 5 per group). Representative images of the back.

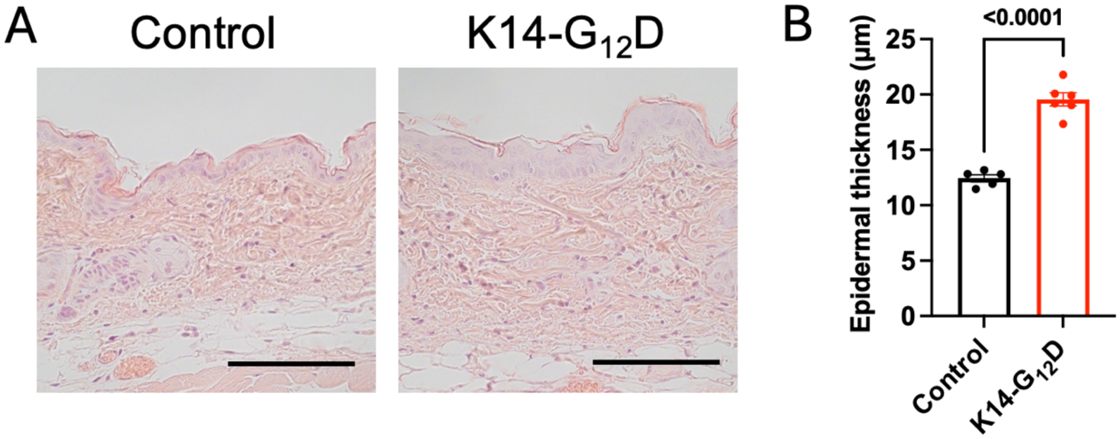

Figure S4. Effect of $G_{12}D$ activation in the absence of wax depilation.

A, Representative HE staining of dorsal skin section. Scale bars: 100 µm.

B, Quantification of epidermal thickness shown in (A) (n = 5–6 per group). Values represent mean ± SEM. Data were analyzed by the two-tailed Student's t-test.

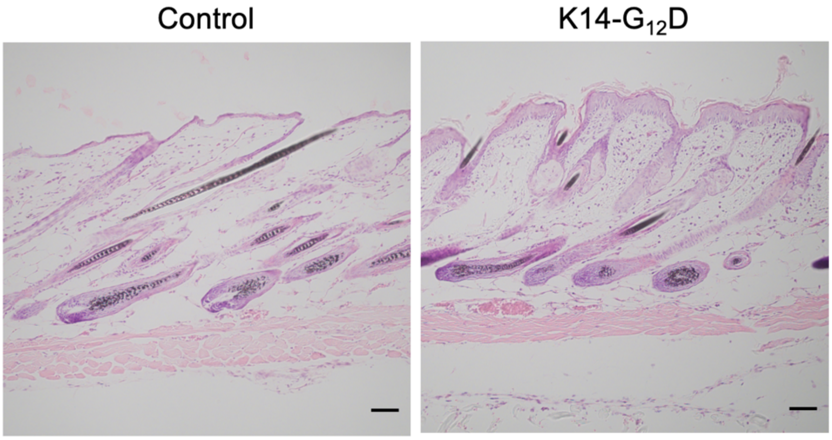

Figure S5. Low magnification images of HE staining of skin sections.

Representative low-magnification images of HE staining are shown. Scale bars: 100 μm.

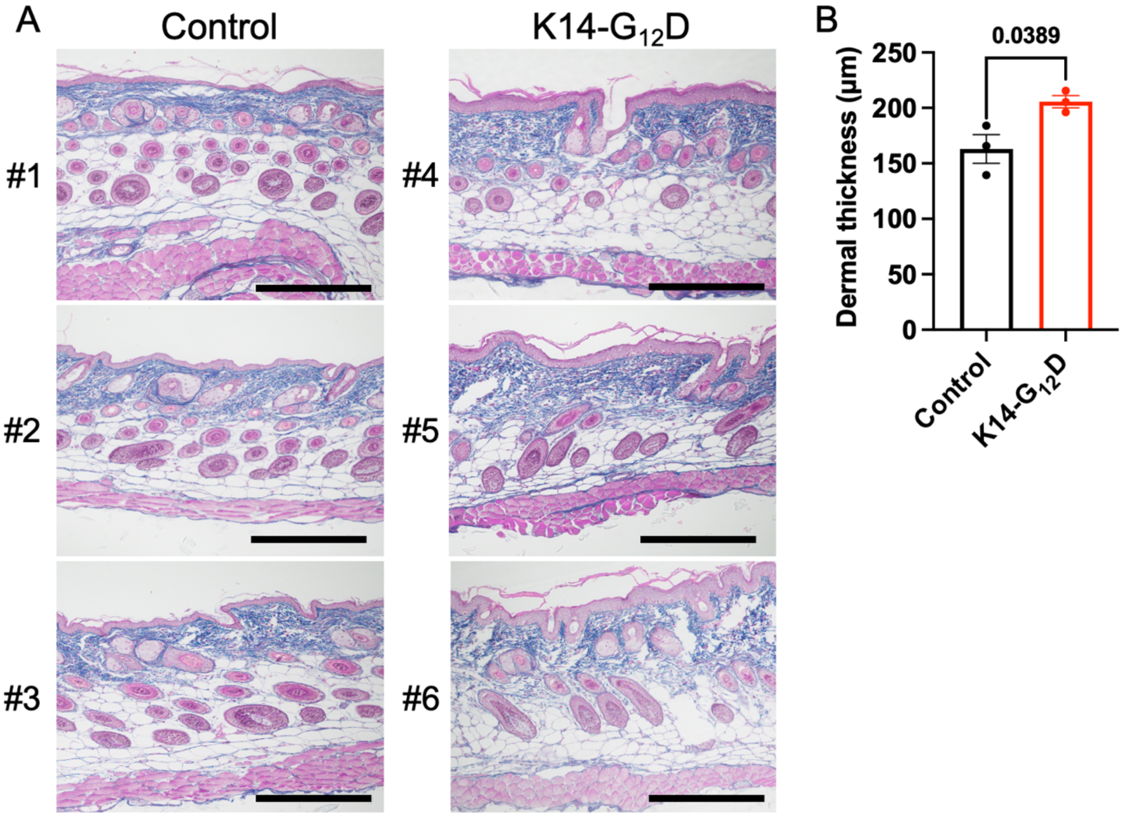

Figure S6. Effect of G_{12}D activation on dermis.

A, Representative Masson trichrome staining of dorsal skin specimens from mice administered daily with CNO (10 mg/kg/day) for 10 days. Scale bars: 100 μm.

B, Quantification of dermal thickness shown in (A) (n = 3 per group). Values represent mean ± SEM. Data were analyzed by the two-tailed Student's t-test.

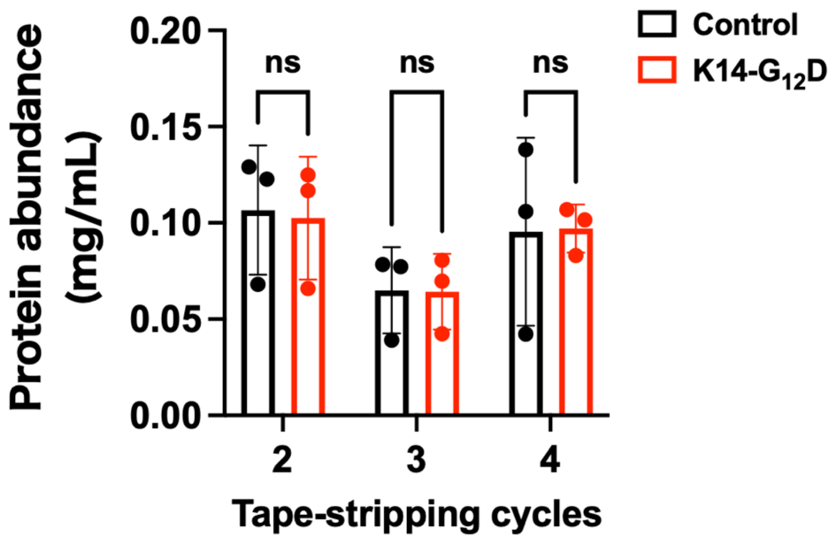

Figure S7. Protein amount in stratum corneum of mouse upon chemogenetic $G_{12}D$ activation.

Proteins on the tape were extracted from the dorsal skin of mice and protein amounts were determined by a BCA method (n = 3 per group).

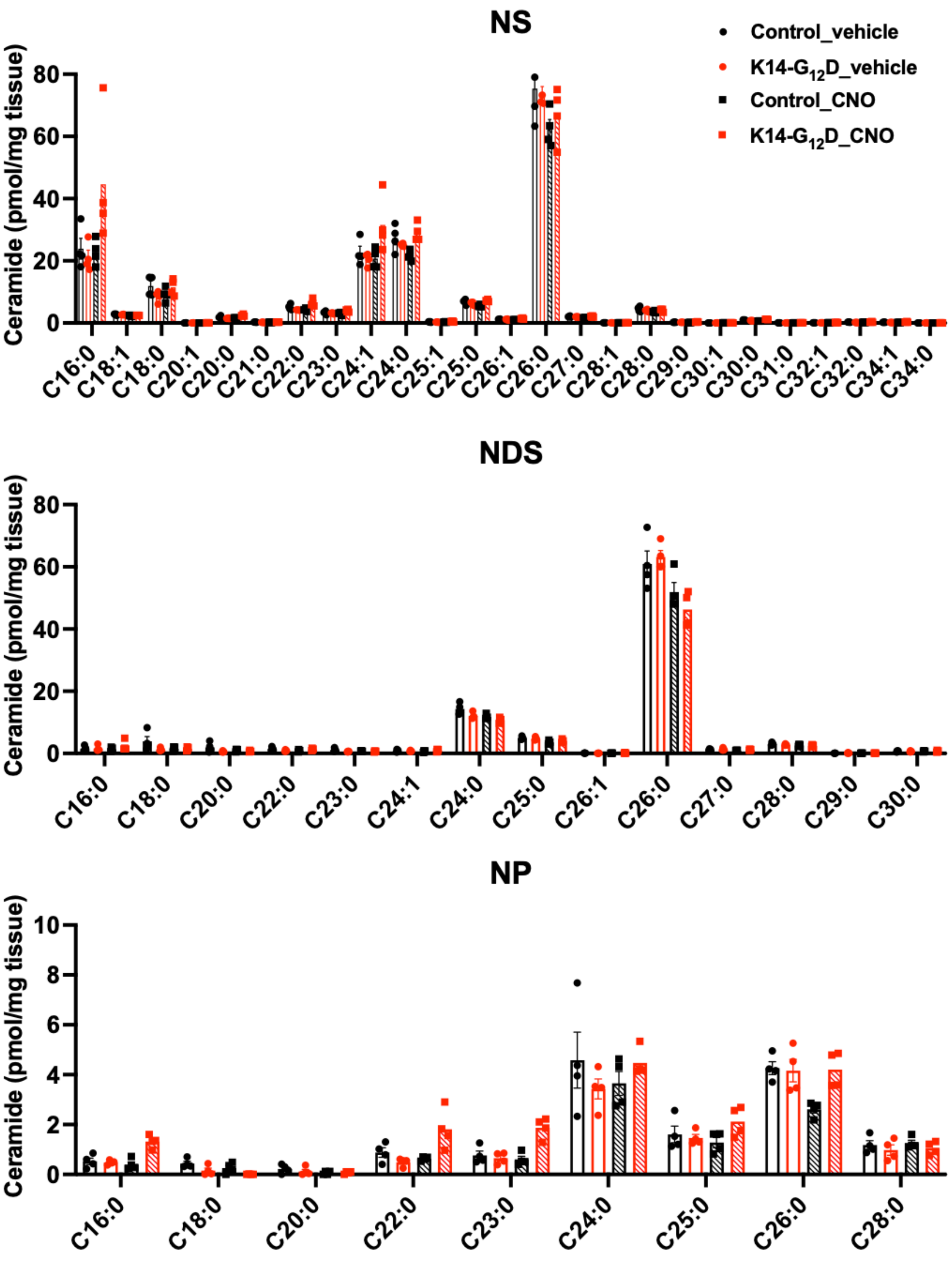

NS
Control_vehicle
K14-G12D_vehicle
Control_CNO
K14-G12D_CNO
Ceramide (pmol/mg tissue)
0
20
40
60
80
C16:0
C18:1
C18:0
C20:1
C20:0
C21:0
C22:0
C23:0
C24:1
C24:0
C25:1
C25:0
C26:1
C26:0
C27:0
C28:1
C28:0
C29:0
C30:1
C30:0
C31:0
C32:1
C32:0
C34:1
C34:0
NDS
Ceramide (pmol/mg tissue)
0
20
40
60
80
C16:0
C18:0
C20:0
C22:0
C23:0
C24:1
C24:0
C25:0
C26:1
C26:0
C27:0
C28:0
C29:0
C30:0
NP
Ceramide (pmol/mg tissue)
0
2
4
6
8
10
C16:0
C18:0
C20:0
C22:0
C23:0
C24:0
C25:0
C26:0
C28:0

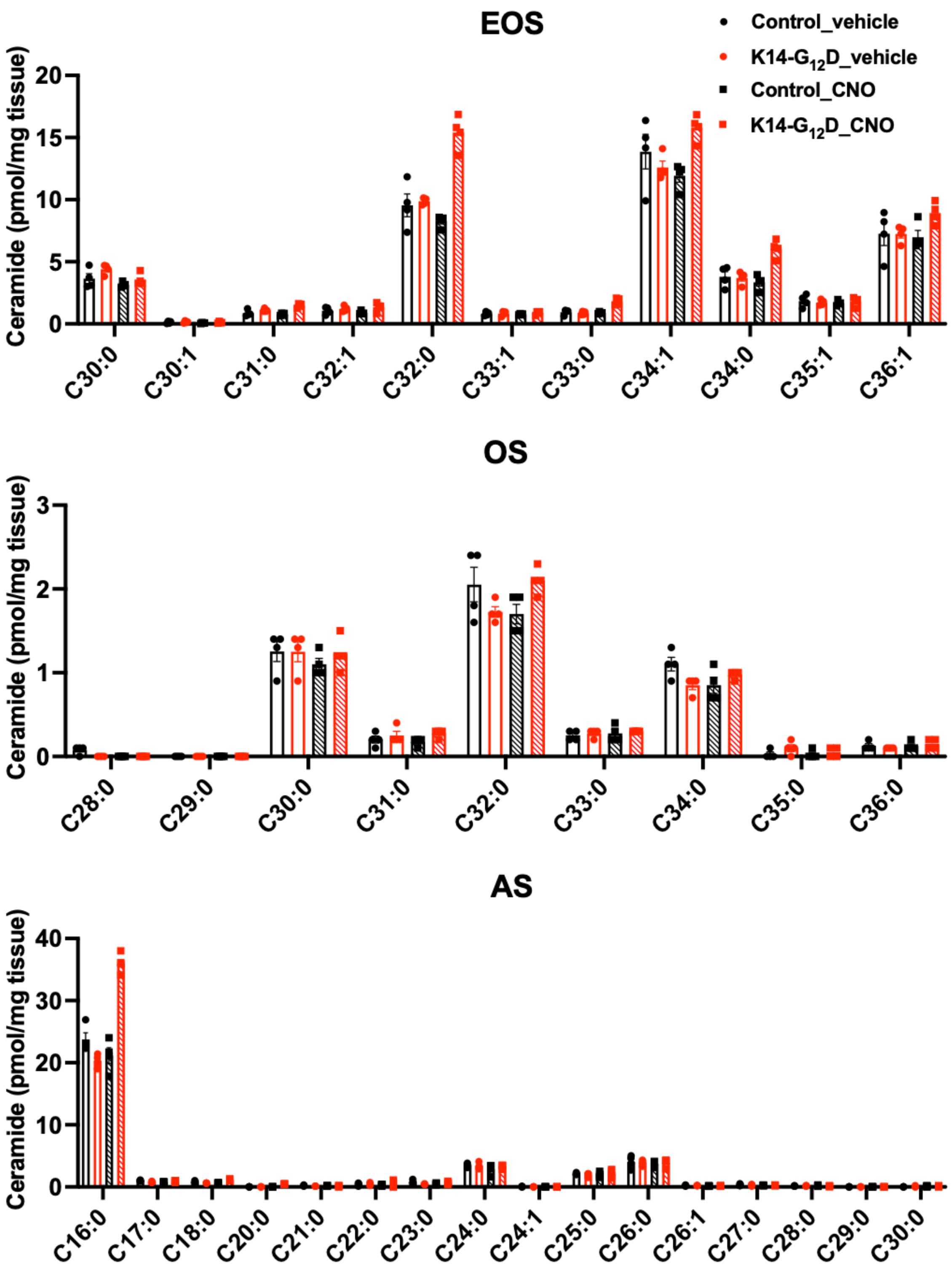
EOS
Control_vehicle
K14-G12D_vehicle
Control_CNO
K14-G12D_CNO
Ceramide (pmol/mg tissue)
20
15
10
5
0
C30:0
C30:1
C31:0
C32:1
C32:0
C33:1
C33:0
C34:1
C34:0
C35:1
C36:1
OS
Ceramide (pmol/mg tissue)
3
2
1
0
C28:0
C29:0
C30:0
C31:0
C32:0
C33:0
C34:0
C35:0
C36:0
AS
Ceramide (pmol/mg tissue)
40
30
20
10
0
C16:0
C17:0
C18:0
C20:0
C21:0
C22:0
C23:0
C24:0
C24:1
C25:0
C26:0
C26:1
C27:0
C28:0
C29:0
C30:0

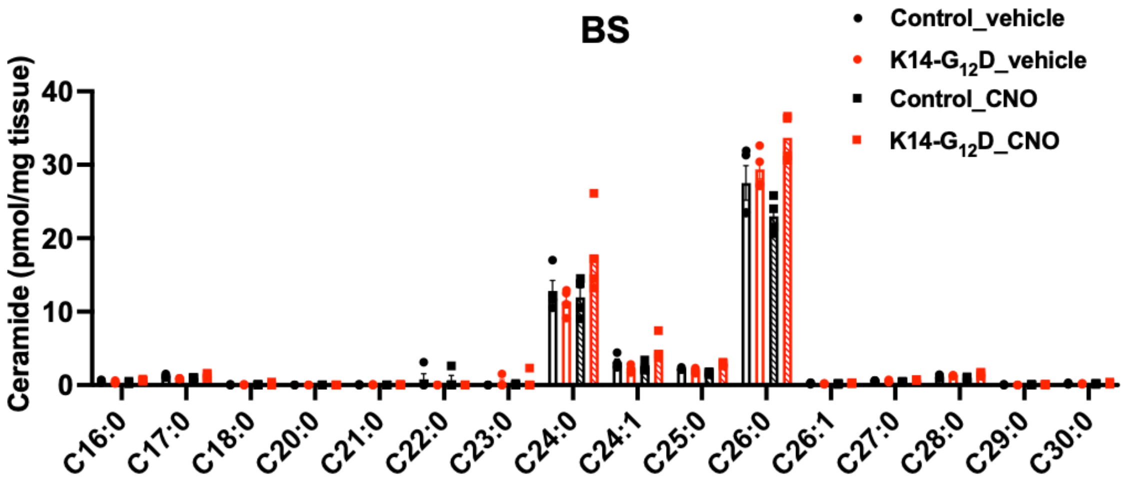

Figure S8. Ceramide profile in mouse skin upon chemogenetic $G_{12}D$ activation. Ceramides were extracted from the dorsal skin samples and quantified by LC/MS/MS analyses (n = 4 per group).

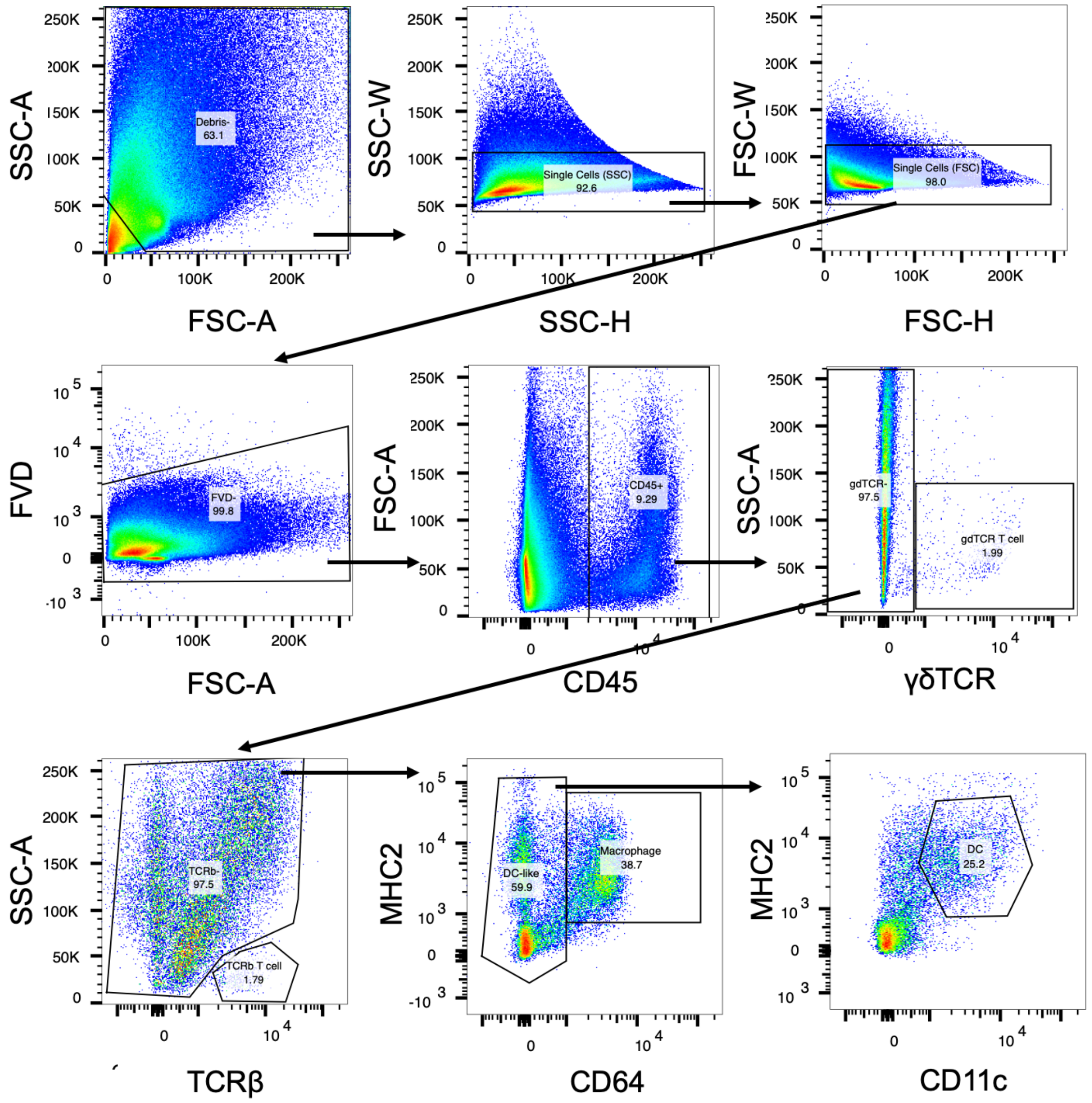

Figure S9. Gating strategy for flow cytometric analysis of immune cell populations in dorsal skin.

Representative gating strategy used for flow cytometric analysis of dorsal skin immune cells. After exclusion of debris, doublets, and dead cells, live $CD45^+$ cells were selected. From this population, γδT cells and $TCR\beta^+$ T cells were identified, and antigen-presenting cell populations were further analyzed based on MHC II, CD64, and CD11c expression to define macrophages and dendritic cells.

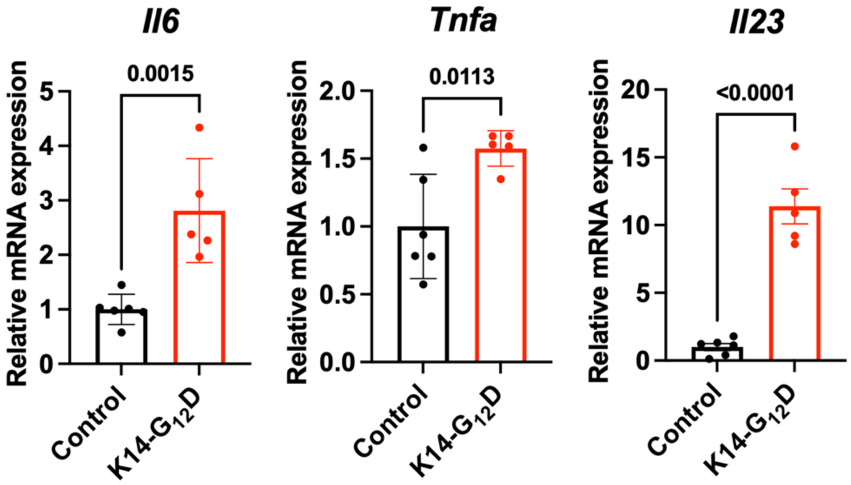

Figure S10. Effect of $G_{12}D$ activation on cytokine expression.

RT-qPCR analyses of the expression of inflammatory cytokines (*Il6*, *Tnfa*, and *Il23*). Normalized fold changes in the expression of the respective genes are shown, with *Hprt* as the reference. Results are shown as means ± SEM (n = 5 or 6). Data were analyzed by the two-tailed Student's t-test.

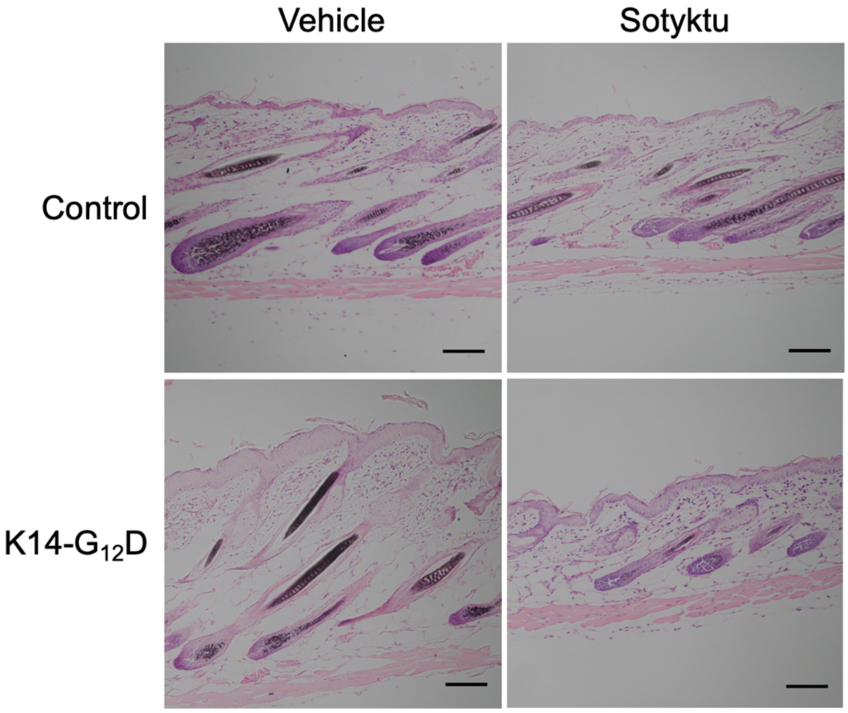

Figure S11. Low magnification images of HE staining of skin sections.

Representative low-magnification images of HE staining are shown. Scale bars: 100 μm.

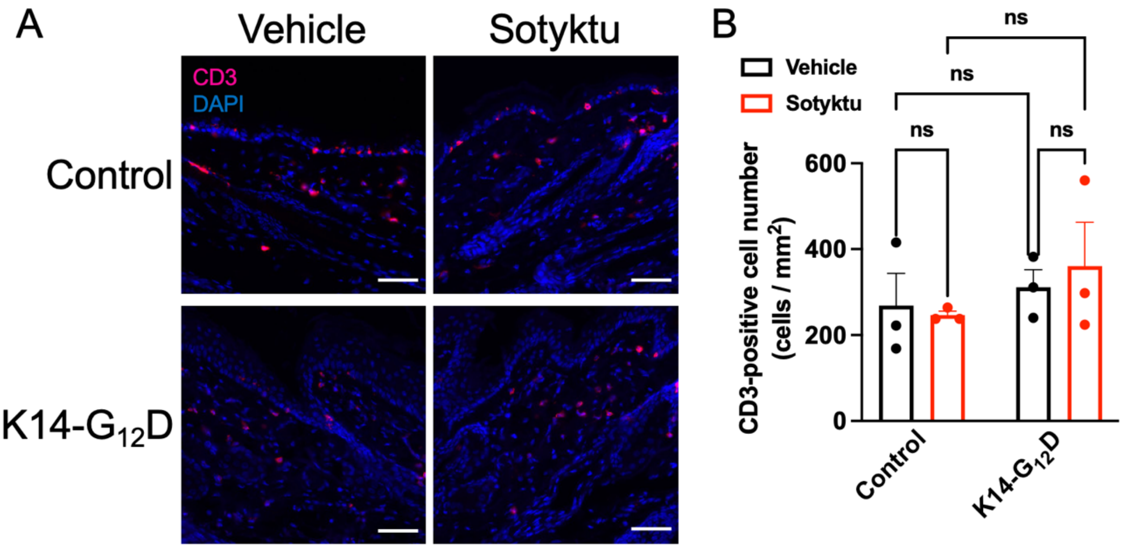

Figure S12. Immunostaining of dorsal skin specimens with anti-CD3 antibody.

A, Representative images of immunostaining for CD3 (*n* = 3 per group). Scale bars: 50 µm.

B, Quantification of CD3-positive cell numbers (*n* = 3). Values represent mean ± SEM. Data were analyzed by the two-way ANOVA.

Table S1. RT-qPCR primers used in this study

Gene	Forward primer (5'→3')	Reverse primer (5'→3')
Flg	ACTCAACCAGTGATAAGGAGGAAG	AGCGATGTCTTGGTCATCTGG
Cldn1	CTTGACCCCCATCAATGC	GTGGTGTTGGGTAAGAGGTTG
Il6	TACCACTTCACAAGTCGGAGGC	CTGCAAGTGCATCATCGTTGTTC
Tnfa	CCACCACGCTCTTCTGTCTAC	AGGGTCTGGGCCATAGAACT
Il23	GCACCAGCGGGACATATGAA	AGACCTTGGCGGATCCTTTG
Gapdh	AGGAGCGAGACCCCACTAAC	CGGAGATGATGACCCTTTTG
Hprt	AGTCCCAGCGTCGTGATTAG	TGATGGCCTCCCATCTCCTT

Table S2. MRM settings for LC/MS/MS analysis of ceramide species

Ceramide Species	Precursor ions (Q1)		Product ion (Q3)	Collision energy (eV)
	$[M–H_2O+H]^+$	$[M+H]^+$		
NDS (d_9-C16:0)		549.5	284.3	20
NDS (C16:0)		540.5	284.3	20
NDS (C18:0)		568.6	284.3	20
NDS (C20:0)		596.6	284.3	20
NDS (C22:0)		624.6	284.3	25
NDS (C23:0)		638.6	284.3	25
NDS (C24:0)		652.6	284.3	32
NDS (C24:1)		650.6	284.3	30
NDS (C25:0)		666.6	284.3	30
NDS (C26:0)		680.7	284.3	30
NDS (C26:1)		678.7	284.3	30
NDS (C27:0)		694.7	284.3	30
NDS (C28:0)		708.7	284.3	30
NDS (C29:0)		722.7	284.3	30
NDS (C30:0)		736.7	284.3	35
NS (d_9-C16:0)	529.4		264.3	20
NS (C16:0)	520.5		264.3	20
NS (C18:0)	548.5		264.3	20
NS (C18:1)	546.5		264.3	20
NS (C20:0)	576.5		264.3	20
NS (C20:1)	574.5		264.3	20
NS (C21:0)	590.6		264.3	25
NS (C22:0)	604.6		264.3	25
NS (C23:0)	618.6		264.3	30
NS (C24:0)	632.6		264.3	30
NS (C24:1)	630.6		264.3	30
NS (C25:0)	646.7		264.3	30
NS (C25:1)	644.7		264.3	30

NS (C26:0)	660.7		264.3	30
NS (C26:1)	658.7		264.3	30
NS (C27:0)	674.7		264.3	30
NS (C28:0)	688.7		264.3	30
NS (C28:1)	686.7		264.3	30
NS (C29:0)	702.7		264.3	30
NS (C30:0)	716.7		264.3	35
NS (C30:1)	714.7		264.3	35
NS (C31:0)	730.7		264.3	35
NS (C32:0)	744.8		264.3	40
NS (C32:1)	742.8		264.3	35
NS (C34:0)	772.8		264.3	40
NS (C34:1)	770.8		264.3	40
NP (d_9-C16:0)		565.5	300.3	25
NP (C16:0)		556.6	300.3	25
NP (C18:0)		584.6	300.3	25
NP (C20:0)		612.6	300.3	25
NP (C22:0)		640.7	300.3	30
NP (C23:0)		654.7	300.3	30
NP (C24:0)		668.7	300.3	30
NP (C25:0)		682.7	300.3	30
NP (C26:0)		696.7	300.3	30
NP (C28:0)		724.8	300.3	35
AS (d_9-C16:0)	545.5		264.3	20
AS/BS (C16:0)	536.5		264.3	20
AS/BS (C17:0)	550.5		264.3	20
AS/BS (C18:0)	564.6		264.3	20
AS/BS (C20:0)	592.6		264.3	20
AS/BS (C21:0)	606.6		264.3	25
AS/BS (C22:0)	620.6		264.3	25
AS/BS (C23:0)	634.6		264.3	25

AS/BS (C24:0)	648.6		264.3	30
AS/BS (C24:1)	646.6		264.3	30
AS/BS (C25:0)	662.6		264.3	30
AS/BS (C26:0)	676.7		264.3	30
AS/BS (C26:1)	674.7		264.3	30
AS/BS (C27:0)	690.7		264.3	30
AS/BS (C28:0)	704.7		264.3	30
AS/BS (C29:0)	718.7		264.3	30
AS/BS (C30:0)	732.7		264.3	35
OS (C28:0)	704.7	722.7	264.3	30
OS (C29:0)	718.7	736.7	264.3	30
OS (C30:0)	732.7	750.7	264.3	35
OS (C31:0)	746.7	764.7	264.3	35
OS (C31:1)	744.7	762.7	264.3	35
OS (C32:0)	760.8	778.8	264.3	40
OS (C32:1)	758.8	776.8	264.3	35
OS (C33:0)	774.8	792.8	264.3	40
OS (C33:1)	772.8	790.8	264.3	40
OS (C34:0)	788.8	806.8	264.3	40
OS (C34:1)	786.8	804.8	264.3	40
OS (C35:0)	802.8	820.8	264.3	40
OS (C35:1)	800.8	818.8	264.3	40
OS (C36:1)	814.8	832.8	264.3	40
EOS (C30:0)	995.0	1013.0	264.3	40
EOS (C30:1)	993.0	1011.0	264.3	40
EOS (C31:0)	1009.0	1027.0	264.3	40
EOS (C32:0)	1023.0	1041.0	264.3	40
EOS (C32:1)	1021.0	1039.0	264.3	40
EOS (C33:0)	1037.0	1055.0	264.3	40
EOS (C33:1)	1035.0	1053.0	264.3	40
EOS (C34:0)	1051.1	1069.1	264.3	40

EOS (C34:1)	1049.1	1067.1	264.3	40
EOS (C35:1)	1063.1	1081.1	264.3	40
EOS (C36:1)	1077.1	1095.1	264.3	45

Table S3. Upregulated GO terms in K14-G_{12}D mice.

Term name	Adjusted *P* value
immune system process	9.97e-25
immune response	1.55e-23
defense response	7.98e-23
keratinization	6.32e-21
response to external stimulus	5.11e-19
biological process involved in interspecies interaction between organisms	1.20e-17
response to other organism	8.31e-17
epidermis development	8.58e-17
response to external biotic stimulus	9.72e-17
epidermal cell differentiation	3.263e-16
keratinocyte differentiation	3.72e-16
response to biotic stimulus	3.88e-16
skin development	4.00e-16
defense response to symbiont	1.27e-14
defense response to bacterium	4.47e-14
regulation of immune system process	5.18e-14
defense response to other organism	5.91e-14
positive regulation of immune system process	2.030e-13
immune effector process	1.51e-12
regulation of immune response	1.64e-12
response to stress	9.88e-12
antimicrobial humoral response	2.99e-11
defense response to Gram-positive bacterium	5.96e-11
positive regulation of immune response	9.94e-11
lipid metabolic process	1.23e-10
inflammatory response	6.14e-10
innate immune response	1.02e-9
T-helper 1 type immune response	1.05e-9

antigen processing and presentation	1.14e-9
response to bacterium	1.46e-9
regulation of adaptive immune response	4.89e-9
antigen processing and presentation of peptide antigen	7.82e-9
leukocyte activation	1.04e-8
epithelial cell differentiation	1.07e-8
leukocyte mediated immunity	1.09e-8
cell activation	2.22e-8
humoral immune response	3.10e-8
regulation of response to external stimulus	5.01e-8
cytokine-mediated signaling pathway	5.61e-8
regulation of immune effector process	7.66e-8
adaptive immune response based on somatic recombination of immune receptors built from immunoglobulin superfamily domains	8.39e-8
response to stimulus	1.73e-7
positive regulation of multicellular organismal process	1.82e-7
positive regulation of response to stimulus	2.82e-7
cell chemotaxis	4.18e-7
cell surface receptor signaling pathway	4.66e-7
regulation of adaptive immune response based on somatic recombination of immune receptors built from immunoglobulin superfamily domains	5.40e-7
lymphocyte mediated immunity	6.52e-7
type II interferon production	7.40e-7
regulation of multicellular organismal process	8.42e-7
regulation of cytokine production	9.72e-7
cytokine production	1.19e-06
regulation of defense response	1.44e-06
T cell activation	1.58e-06
epithelium development	2.18e-06

lymphocyte activation	2.39e-06
mononuclear cell differentiation	2.39e-06
lymphocyte differentiation	2.79e-06
regulation of T-helper 1 type immune response	3.29e-06
cellular response to chemical stimulus	4.00e-06
chemotaxis	4.50e-06
taxis	5.03e-06
positive regulation of cytokine production	5.45e-06
antibacterial humoral response	6.11e-06
regulation of type II interferon production	6.39e-06
response to peptide	6.97e-06
leukocyte differentiation	7.52e-06
regulation of response to stimulus	8.13e-06
leukocyte chemotaxis	1.09e-05
response to cytokine	1.33e-05
leukocyte activation involved in immune response	2.09e-05
cellular response to cytokine stimulus	2.30e-05
cell activation involved in immune response	2.38e-05
leukocyte migration	3.22e-05
cell killing	3.34e-05
positive regulation of immune effector process	3.84e-05
non-canonical NF-kappaB signal transduction	4.09e-05
regulation of T cell activation	4.74e-05
positive regulation of adaptive immune response	5.09e-05
antigen processing and presentation of peptide antigen via MHC class I	5.74e-05
T cell differentiation	6.08e-05
regulation of response to biotic stimulus	7.27e-05
locomotion	8.81e-05
cell differentiation	8.84e-05
cellular developmental process	8.96e-05

tissue development	9.21e-05
positive regulation of gene expression	9.55e-05
adaptive immune response	9.98e-05
positive regulation of response to external stimulus	0.000103
regulation of leukocyte cell-cell adhesion	0.000111
leukocyte cell-cell adhesion	0.0001290
regulation of T cell mediated immunity	0.000133
regulation of interleukin-1 production	0.000159
interleukin-1 production	0.000159
leukocyte mediated cytotoxicity	0.000194
regulation of leukocyte activation	0.000195
establishment of skin barrier	0.00021
negative regulation of immune response	0.000222
positive regulation of leukocyte cell-cell adhesion	0.000237
regulation of interleukin-6 production	0.000264
interleukin-6 production	0.000264
regulation of non-canonical NF-kappaB signal transduction	0.000298
regulation of cell activation	0.000354
positive regulation of T cell activation	0.000357
regulation of lymphocyte mediated immunity	0.000360
programmed cell death	0.000497
cell death	0.000497
positive regulation of defense response	0.000632
antigen processing and presentation of exogenous peptide antigen	0.000708
antimicrobial humoral immune response mediated by antimicrobial peptide	0.000708
positive regulation of biological process	0.000733
regulation of lymphocyte activation	0.000776
regulation of leukocyte mediated cytotoxicity	0.000793
positive regulation of signal transduction	0.000873

sphingolipid metabolic process	0.000971
positive regulation of interleukin-6 production	0.000982
positive regulation of non-canonical NF-kappaB signal transduction	0.000994
regulation of defense response to bacterium	0.00109
response to oxygen-containing compound	0.00119
regulation of antimicrobial humoral response	0.00122
granulocyte chemotaxis	0.00136
lipid biosynthetic process	0.00159
apoptotic process	0.00165
positive regulation of interleukin-1 production	0.00180
developmental process	0.00189
positive regulation of antigen processing and presentation	0.00207
host-mediated modulation of intestinal microbiota composition	0.00207
small molecule metabolic process	0.00210
multicellular organismal process	0.00212
sphingoid metabolic process	0.00212
positive regulation of cell activation	0.00213
cell-cell adhesion	0.00215
regulation of leukocyte mediated immunity	0.00223
lipid catabolic process	0.00226
activation of immune response	0.00230
positive regulation of cell-cell adhesion	0.00231
regulation of cell killing	0.00242
regulation of antibacterial peptide production	0.00250
T cell activation involved in immune response	0.00253
response to virus	0.00256
regulation of leukocyte migration	0.00285
antigen processing and presentation of exogenous antigen	0.00294
immune response-regulating signaling pathway	0.00297

T cell mediated immunity	0.00308
regulation of cell-cell adhesion	0.00334
T cell migration	0.00357
negative regulation of type II interferon production	0.00363
small molecule biosynthetic process	0.00383
hemopoiesis	0.00385
negative regulation of immune system process	0.00398
positive regulation of leukocyte activation	0.00424
monocarboxylic acid biosynthetic process	0.00431
cell migration	0.00440
positive regulation of defense response to bacterium	0.00510
positive regulation of cellular process	0.00523
regulation of inflammatory response	0.00535
immune response-activating signaling pathway	0.00604
signal transduction	0.00623
signaling	0.00627
T-helper 1 cell differentiation	0.00654
myeloid leukocyte migration	0.00682
alpha-beta T cell activation involved in immune response	0.00751
neutrophil chemotaxis	0.00751
anatomical structure development	0.00795
positive regulation of cell communication	0.00910
positive regulation of production of molecular mediator of immune response	0.00921
positive regulation of signaling	0.00926
positive regulation of adaptive immune response based on somatic recombination of immune receptors built from immunoglobulin superfamily domains	0.00998
granulocyte migration	0.00998
negative regulation of multicellular organismal process	0.0101
sebaceous gland cell differentiation	0.0101

cell communication	0.0102
multicellular organismal-level homeostasis	0.0108
regulation of antimicrobial peptide production	0.0113
cell adhesion	0.0121
positive regulation of chemokine production	0.0124
T cell differentiation involved in immune response	0.0147
defense response to virus	0.0152
positive regulation of cytokine production involved in immune response	0.0163
diol metabolic process	0.0165
modulation of process of another organism	0.0167
CD4-positive, alpha-beta T cell differentiation	0.0167
positive regulation of response to biotic stimulus	0.0168
regulation of interleukin-1 beta production	0.0181
interleukin-1 beta production	0.0181
regulation of innate immune response	0.0184
regulation of cell population proliferation	0.0192
positive regulation of intracellular signal transduction	0.0194
monocarboxylic acid metabolic process	0.0195
positive regulation of ERK1 and ERK2 cascade	0.0197
T-helper cell differentiation	0.0201
antibacterial peptide production	0.0202
acetylcholine receptor signaling pathway	0.0204
positive regulation of T-helper 1 type immune response	0.0206
positive regulation of lymphocyte activation	0.0210
production of molecular mediator of immune response	0.0215
tumor necrosis factor production	0.0217
regulation of tumor necrosis factor production	0.0217
response to chemical	0.0239
cellular response to acetylcholine	0.0249
positive regulation of MAPK cascade	0.0265

tumor necrosis factor superfamily cytokine production	0.0265
regulation of tumor necrosis factor superfamily cytokine production	0.0265
negative regulation of adaptive immune response based on somatic recombination of immune receptors built from immunoglobulin superfamily domains	0.0284
proteolysis	0.0288
unsaturated fatty acid metabolic process	0.0329
interleukin-18-mediated signaling pathway	0.0332
fatty acid biosynthetic process	0.0344
regulation of antigen processing and presentation	0.0363
sphingosine metabolic process	0.0363
positive regulation of T cell mediated immunity	0.0377
regulation of response to stress	0.0400
positive regulation of antibacterial peptide production	0.0401
antigen processing and presentation of peptide antigen via MHC class Ib	0.04016
regulation of apoptotic process	0.0414
cell motility	0.0420
alpha-beta T cell differentiation	0.0426
neutrophil migration	0.0434
positive regulation of lymphocyte mediated immunity	0.0457
host-mediated perturbation of symbiont process	0.0476
myeloid cell activation involved in immune response	0.0476
cellular response to stimulus	0.0490
negative regulation of adaptive immune response	0.0494
